# The promise and pitfalls of cross-partisan conversations for reducing affective polarization: Evidence from randomized experiments

**DOI:** 10.1126/sciadv.abn5515

**Published:** 2022-06-22

**Authors:** Erik Santoro, David E. Broockman

**Affiliations:** 1 Department of Psychology, Stanford University, Stanford, CA 94305, USA.; 2 Charles and Louise Travers Department of Political Science, University of California, Berkeley, Berkeley, CA 94720, USA.

## Abstract

Organizations, activists, and scholars hope that conversations between outpartisans (supporters of opposing political parties) can reduce affective polarization (dislike of outpartisans) and bolster democratic accountability (e.g., support for democratic norms). We argue that such conversations can reduce affective polarization but that these effects are likely to be conditional on topic, being especially likely if the conversations topics avoid discussion of areas of disagreement; usually not persist long-term; and be circumscribed, not affecting attitudes toward democratic accountability. We support this argument with two unique experiments where we paired outpartisan strangers to discuss randomly assigned topics over video calls. In study 1, we found that conversations between outpartisans about their perfect day dramatically decreased affective polarization, although these impacts decayed long-term. Study 2 also included conversations focusing on disagreement (e.g., why each supports their own party), which had no effects. Both studies found little change in attitudes related to democratic accountability.

## Teaser

Cross-partisan conversations can reduce affective polarization, but effects do not persist long-term or spill over.

## INTRODUCTION

Americans who support Democrats and who support Republicans have never disliked each other more, a phenomenon known as affective polarization (*1*). They cut short Thanksgiving dinners with each other (*2*), avoid dating each other (*3*), and discriminate against each other (*4*). Scholars worry about these social consequences of affective polarization as well as its potential negative consequences for democracy. For example, scholars worry that affective polarization “weaken[s]...willingness to punish one’s own party’s politicians” [(*5*), p. 50], “increases partisans’ willingness to conform to their party’s policy positions” [(*6*), p. 142], and reduces support for legislative bipartisanship (*7*).

Inspired by theories of intergroup contact, these concerns for society and democracy have led a number of organizations, activists, and scholars to embrace cross-partisan conversations as a strategy for reducing affective polarization—and, in turn, improving democratic accountability. For instance, a recent review calls for research on whether such conversations “could potentially reduce partisan animus” (*6*). Similarly, a recent *New York Times* report details a bevy of “classes, apps and message boards...trying to bridge the divide between the left and the right, one conversation at a time” (*8*). More generally, scholars and practitioners alike have proposed a variety of other strategies to address affective polarization—such as mandatory national service (*9*)—premised in large part on the notion that getting everyday Democrats and Republicans to interact face-to-face would reduce affective polarization. Many further expect that any such reductions in affective polarization would have positive downstream effects for democratic accountability (e.g., increasing support for legislative bipartisanship or increasing support for democratic norms). Understanding the impacts of cross-partisan conversations and how best to conduct them therefore has the potential to unlock both important practical insights and theoretical lessons.

Here, we provide a focused test of the hypothesis that undergirds many scholars’ and practitioners’ proposals for reducing affective polarization: that getting Democrats and Republicans to talk face-to-face would reduce affective polarization and, in turn, have positive consequences for democracy. Drawing on theories of intergroup contact [e.g., (*10*)], we argue that conversation topics that encourage discussion of areas of agreement instead of disagreement have the potential to meaningfully reduce affective polarization.

At the same time, we also theorize limits to the effects of these conversations. Building on theories of persuasive communication from political science and psychology, we argue that the effects of these conversations are likely not to persist long-term. Furthermore, in contrast to many scholars’ hopes that strategies that reduce affective polarization would have positive downstream effects for attitudes toward democracy [e.g., (*7*)], we argue that the effects of these conversations should be circumscribed to intergroup attitudes and have limited effects on attitudes related to democratic accountability.

We present evidence in support of these arguments from a series of unique experiments where we paired Democrats and Republicans in face-to-face conversations over video calls, enabled by custom software we developed. In study 1, we conduct a focused test of the hypothesis that cross-partisan conversations would reduce affective polarization. To do so, we paired participants with an outpartisan participant to discuss a topic designed to elicit discussion of shared experiences, their perfect day. In a Placebo condition, participants were not told that they were talking with an outpartisan; in the Perfect Day condition, participants were informed just before the conversation began that their conversation partner was an outpartisan. In study 2, we replicated these results and tested our argument regarding the importance of whether the conversation topic encouraged discussion of areas of disagreement versus agreement. To do so, we recruited a more representative sample of participants and randomized them into four conditions, two that replicated study 1 as well as two additional conditions inspired by research regarding the potential pitfalls of explicit discussions of areas where individuals disagree [e.g., (*11*)]: an Inparty Strengths condition asking participants to talk about what they liked about their party and an Outparty Flaws condition asking participants to talk about what they disliked about the other party. Table 1 summarizes the conditions.

**Table 1.** Conditions in studies 1 and 2.

**Condition**	**Conversation prompt**	**Informed partner was** **outpartisan?**	**Included in study 1?**	**Included in study 2?**
Placebo	Discuss perfect day (Fast Friends paradigm)	–	✓	✓
Perfect Day	Discuss perfect day (Fast Friends paradigm)	✓	✓	✓
Inparty Strengths	Discuss diverging partisan preferences (why like inparty)	✓	–	✓
Outparty Flaws	Discuss diverging partisan preferences (why do not like outparty)	✓	–	✓

Notes: Participants were always matched with outpartisans but were only informed that their conversation partner was an outpartisan in the three treatment conditions (all conditions but Placebo).

After the conversations in both studies, we asked about items tapping intergroup attitudes (e.g., affective polarization), outcomes relevant to democratic accountability (e.g., testing for party-line voting), and posited mechanisms. In study 1, we also conducted a follow-up survey 3 months after the conversations to see whether the effects persisted long-term.

In both studies, cross-partisan conversations about their perfect day caused very large decreases in affective polarization—enough to reverse two decades worth of increase. Moreover, supporting our argument that encouraging participants to discuss areas of disagreement can be counterproductive, study 2 found that discussing their diverging partisan preferences diminished the effect of these conversations on affective polarization to near zero.

In the context of the literature (reviewed below), several features make our experiments notable: Our experiments involve real, face-to-face interactions between real outpartisans (not confederates); we test the effects of novel conversational topics, varying whether participants were encouraged to discuss disagreement instead of agreement; our research design allows us to isolate the causal effects of these conversations, not containing other elements; we examine often-hoped-for effects on attitudes relevant to democratic accountability (in addition to intergroup attitudes); and we examine long-run effects, a rare feature of experiments on any form of intergroup contact (*10*, *12*).

Our findings are important for at least three reasons. First, our findings confirm the promise of cross-partisan conversations: We found large declines in affective polarization despite that many of the conditions traditionally understood to be necessary for contact to reduce intergroup animosity were not met in this setting. This is all the more surprising given that the intervention was only a brief conversation between untrained laypeople. Second, we show that what is talked about matters. Our results suggest that explicit discussions of areas of disagreement may undermine the salutary effects of intergroup contact. This result complements other recent research on the benefits of highlighting similarities between outpartisans [e.g., (*13*–*16*)] to suggest that, to the extent that intergroup conversations discuss topics related to group differences (e.g., politics or partisanship), such discussions may wish to focus on similarities between individuals or between groups. Last, our findings indicate that cross-partisan conversations have at least two limitations. First, the observed reduction in affective polarization decays in the long-term (i.e., after 3 months)—which was not a foregone conclusion in light of other studies that have found that brief conversations can durably improve intergroup attitudes from a week (*15*) to at least 3 months [e.g., (*17*)]. Second, we did not find robust improvements in outcomes relevant to democratic accountability, despite that many researchers have hoped interventions to reduce affective polarization would have such effects [e.g., (*6*, *7*)].

### Theoretical perspectives

Theoretically, the effects of cross-partisan conversations are not clear ex ante.

#### Should we expect effects at all?

Should we expect cross-partisan conversations to improve intergroup attitudes (e.g., affective polarization) at all? It may seem obvious that they should: At one level, the contact hypothesis suggests that contact between outgroups can lead people to generalize from experiences with a particular outgroup member toward improved intergroup attitudes toward the group in general (*18*–*20*). Moreover, even brief interventions involving humanizing narratives about outgroup members can durably improve intergroup attitudes [e.g., (*17*)]. Consistent with this work, we theorized that brief, positive interactions between outpartisans would reduce affective polarization. (We use the terms “intergroup attitudes,” “partisan animus,” or “affective polarization” rather than “prejudice” throughout because of the fundamental differences between prejudice in the context of a choice-based identity, such as political partisanship, compared to prejudice in the context of immutable identities with sociocultural power and status inequities, such as race and ethnicity, sexuality, etc.)

However, this is by no means obvious, as some literature also suggests that certain conditions may be necessary for intergroup contact to reduce partisan animus: equal status, intergroup cooperation, common goals, and support from authorities. These conditions may be difficult to meet in one-off conversations between outpartisan strangers. Moreover, the fact that individuals often cut short interactions with outpartisans (*2*) suggests that such contact may be unpleasant and so only worsen intergroup attitudes [(*21*, *22*); although see (*23*)].

Despite this theoretical ambiguity, organizations and activists are investing tremendous effort into advocating for and implementing a variety of interventions that heavily feature cross-partisan conversation. For example, interventions intended to reduce affective polarization that range from the notion of mandatory national service to one-off “bridging” workshops all substantially center face-to-face contact and conversation between everyday Democrats and Republicans.

Our work conducts a focused test of the potential for face-to-face contact between Democrats and Republicans to reduce affective polarization: What is the effect of simply having a conversation with an outpartisan stranger? Understanding this question would help shed light on the hypothesis that undergirds a wide variety of interventions posited to reduce affective polarization. Understanding this question is also of theoretical import: There is surprisingly little research that examines the long-run effects of randomly assigned intergroup contact of any sort [see (*10*, *12*)].

#### Which conversation topics are most effective?

We argue that there will be effects of cross-partisan conversations but that these effects will be conditional on the topics discussed. In contexts such as politics where there are salient divides that are perceived to separate the groups (e.g., views toward politicians or on issues), one possibility is that contact can reduce affective polarization by highlighting unexpected similarities or areas of agreement between members of the groups. Existing research has already found that information regarding political similarities between partisans’ demographics or views on issues can reduce affective polarization [e.g., (*14*, *16*)], and so conversations that provide information about such similarities should reduce affective polarization. To test this, our experiments created a situation in which participants would have the opportunity to identify similarities, namely, participants discussed their perfect day, a topic shown to quickly develop rapport and identify similarities (*24*). We expected that even such a “minimal” cross-partisan could reduce partisan animus.

At the same time, many practitioners (see, e.g., livingroomconversations.org, braverangels.org, and https://openmindplatform.org/) encourage cross-partisan conversations to focus on areas of disagreement. Naturally occurring cross-partisan conversations no doubt often dwell on disagreements as well. We therefore chose to also study conversations that encouraged participants to focus on areas of disagreement, as their effects are even more ambiguous on the basis of existing research. In particular, research in other contexts suggests that discussing areas of disagreement between groups might backfire by increasing the salience of group divisions (*11*). On the basis of this research, such salutary effects of cross-partisan contact should be conditional on whether the conversations avoid encouraging discussion of areas where individuals disagree. To test this, our second study included two conditions that explicitly instructed participants to discuss their diverging partisan preferences, in an attempt to encourage participants to discuss areas of disagreement.

#### Should we expect positive downstream effects for democratic accountability?

Practitioners and prior literature alike [for review, see (*25*)] widely expect that interventions that reduce affective polarization—as we show that cross-partisan conversations do—would bolster democratic accountability. Nevertheless, little prior evidence has demonstrated this common assumption. Furthermore, prior research has largely neglected to articulate a theoretical argument regarding why this might happen [for review, see (*25*)]. Were this to occur, we see the likeliest causal chain to be as follows. First, participants’ attitudes toward outparty voters would need to change. Then, this change in attitudes toward outparty voters could generalize to participants’ attitudes toward outparty elites. Last, this change in attitude toward outparty elites could generalize toward respondents’ willingness to vote in a bipartisan manner or support for democratic norms. We find this line of reasoning unlikely, particularly the second link in this causal chain, as research finds that attitudes toward voters are distinct from attitudes toward party elites (*26*). We therefore expect the effects of cross-partisan conversations to be circumscribed to intergroup attitudes, especially absent any attempt to explicitly link intergroup attitudes to attitudes relevant to democratic accountability.

#### Should reductions in partisan animus last in the long-term?

Last, even if cross-partisan conversations had immediate impacts on partisan animus, it is not obvious whether these impacts would endure. Paluck and coauthors (*10*, *12*) have authored recent reviews documenting that, despite the voluminous literature on intergroup contact, relatively few experiments have measured the long-term effects of randomly assigned intergroup contact. On the one hand, several studies find that a brief conversation with a trained canvasser can have impacts that endure for at least 3 months [e.g., (*17*)]. Importantly, Levendusky and Stecula (*15*) similarly find that the effects of a multipronged intervention including cross-partisan conversation last for at least 1 week. However, interventions such as that studied by (*17*) contain components explicitly designed to lead their effects to persist, leaving it unclear theoretically whether cross-partisan conversations between laypeople would have these effects. Because such conversations are unlikely to provide new information that citizens effortfully process (*27*) absent explicit instructions that lead them to do so, and because citizens, following the intervention, reenter their traditional social contexts that fostered polarization in the first place, we argue that such conversations are typically likely to have effects that do not persist in the long-term.

### Existing evidence

Despite conflicting theoretical expectations regarding the likely effects of cross-partisan conversations and the importance of understanding them, existing empirical research directly on the topic of cross-partisan conversations is also somewhat ambiguous, not speaking to the key claims our argument makes. We review a small number of relevant existing studies in more detail in the Supplementary Materials (part C), categorizing relevant studies into four categories.

First, as we review there, only one other study has examined the impacts of cross-partisan conversations directly. In an important paper, Rossiter (*28*) studies text-based cross-partisan conversations. Our study builds on this work in several ways: We measure the effect of partisans discussing a topic centering on areas where individuals disagree, and compare this to the effect of discussing a topic that facilitates agreement; we measure effects on outcomes relevant to democratic accountability; we measure long-run effects; and our conversations take place face-to-face, over video calls instead of text, making them a better proxy of a real-world, scalable intervention.

There are also several studies, listed in table S7, which study interventions that include both conversations with outpartisans and other components. These studies are largely interested in the effects of these interventions, not cross-partisan conversations; accordingly, they all manipulate multiple components beyond cross-partisan conversations, leaving the impacts of cross-partisan conversations ambiguous. Levendusky and Stecula (*15*) show that participants in politically heterogeneous conversation groups who discuss an article about the fact that there is a surprising commonality across partisans experience an improvement in affective polarization and other intergroup attitudes relative to nonpolitical groups who read a nonpolitical article. Although understanding the effects of this compound treatment is of value to practitioners and researchers alike, it is not clear how much of its effects were driven by reading and then discussing an article highlighting similarities between the two parties.

Last, several studies reviewed in table S8 study vicarious, imagined, simulated, or self-reported contact between outpartisans. These studies provide valuable insight and underscore scholars’ interest in the potential of cross-partisan conversations, but do not study how real outpartisans interact with each other in real conversations. In addition, none of these studies examine long-run effects, effects on attitudinal polarization, or effects on democratic accountability. There are also several studies tangentially related to affective polarization or interpersonal conversation we nonexhaustively review in table S9, none of which examine the effects of cross-partisan conversations on affective polarization or democratic accountability.

### Studying cross-partisan conversations experimentally

We conducted two studies that experimentally investigate the effects of cross-partisan conversation on affective polarization and other outcomes. In both studies, we first recruited participants (from sources detailed in Materials and Methods) to a screener survey. For participants who expressed interest and passed other requirements, we invited them to a follow-up survey at a set time. In the follow-up survey, we relied on custom software we developed to pair participants with another outpartisan participant in real time. This software also randomly assigned each dyad (i.e., pair of participants) a conversation topic, which varied by study (see Table 1). Participants were then automatically directed to a video chatroom with their paired outpartisan participant and informed of their randomly assigned topic. After participants spoke, we asked a series of outcome variables. In study 1, we also conducted a long-term follow-up survey. See Materials and Methods for more details on the experimental procedures.

## RESULTS

### Study 1: Do cross-partisan conversations reduce affective polarization?

Study 1 provides our first investigation into the effects of cross-partisan conversations. This study was an attempt to conduct a focused test of the idea that undergirds a wide variety of proposed interventions to reduce affective polarization: that fostering conversations between outpartisans would reduce affective polarization and, in turn, improve outcomes related to democratic accountability. To this end, study 1 instructed participants to discuss a topic designed to elicit discussion of shared experience and thereby help them find similarity between outpartisans: discussing their perfect day (*24*).

Study 1 compared a conversation with an outpartisan about their perfect day (Perfect Day) to a placebo condition (Placebo) where participants discussed the same topic but did not know they were talking with an outpartisan. The experimental manipulation in study 1 is therefore whether participants were aware that they were talking with an outpartisan, given that a conversation began. The conversation topic in every case was “What does the ‘perfect day’ look like to you?” (*24*). The website also told all participants “You will be matched with someone.” If participants had been randomly assigned to the Perfect Day condition, the sentence went on to say “who feels closer to the [OUTPARTY] party. (You told us you feel closer to the [INPARTY] party.)” Participants in the Placebo condition were not told this. This allowed us to hold constant the ability of participants to be matched with an outpartisan given the specific time at which they appeared and for their computer software to be working. Materials and Methods provides further details on study 1’s preregistration, design, analytical approach, exclusions, and outcome variables.

#### Sample characteristics

*N* = 7756 participants completed the screener, of whom we invited *N* = 4506 to the conversation survey. *N* = 986 began the conversation survey and entered the conversation room. Last, *N* = 478 participants were able to successfully begin a conversation, of whom *N* = 218 were in the Placebo group and *N* = 260 were in the Perfect Day group. Table S1 shows the demographics of the sample at each stage and a balance check. The Placebo conversations lasted a median of 11.7 min; the Perfect Day conversations lasted a median of 12.0 min.

We see some evidence that individuals assigned to the Perfect Day group may have been slightly more likely to begin a conversation (*P* = 0.055) and to return to the follow-up survey (*P* = 0.048), but a test for differential attrition by covariates was insignificant, as assessed by a joint hypothesis test on the interactions between covariates and treatment assignment on a regression predicting attrition (*29*) (*P* = 0.28). A balance check finds that the groups who began a conversation in each condition were generally similar on baseline political covariates (see table S1). This balance check did find that men were slightly overrepresented in the Perfect Day condition, perhaps indicating that men were especially interested in having the Perfect Day relative to the Placebo conversation; however, gender is not a significant predictor of our outcomes, and we find similar effect estimates for men and women on the items where we did find effects.

#### Estimated treatment effects

As described in Materials and Methods, we estimate the treatment effects of the Perfect Day condition relative to the Placebo condition using ordinary least squares regression with clustered standard errors (by dyad) and preregistered pretreatment covariates. We also adjust the *P* values we present for multiple comparisons.

**Fig. 1.**
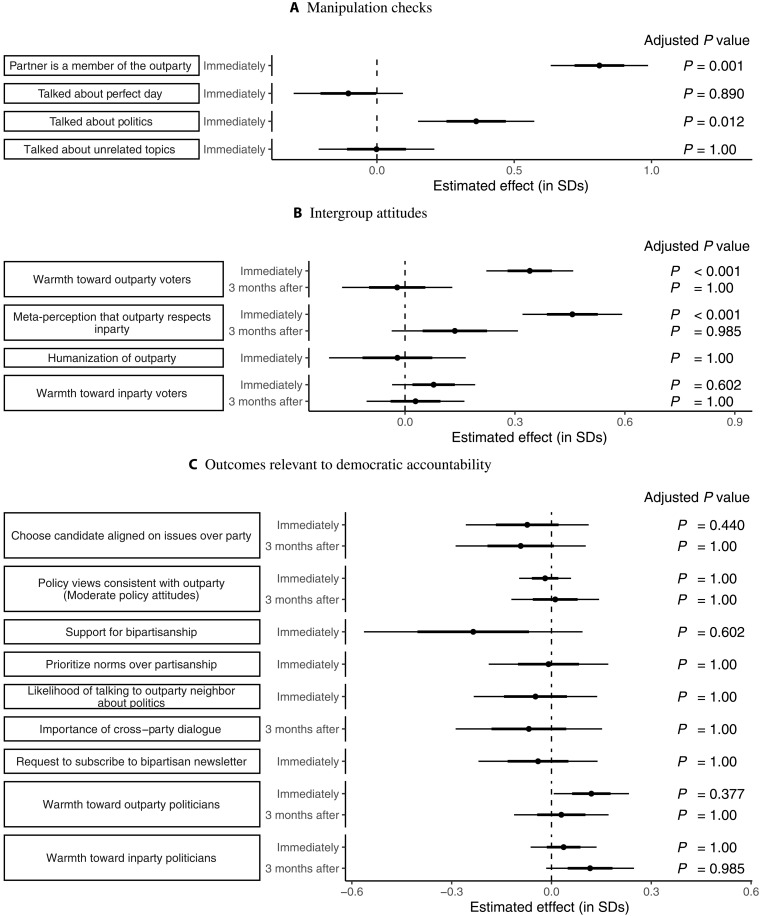
Study 1 results. Notes: Points show the estimated effects of the Perfect Day condition (relative to the Placebo condition) in study 1. Standard errors (thick lines) and 95% confidence intervals (thin lines) surround the point estimates. Adjusted *P* values are across all tests besides the primary outcomes using the procedure outlined in (*30*). See table S3 for numerical results. (**A**) Manipulation checks. (**B**) Intergroup attitudes. (**C**) Outcomes relevant to democratic accountability.

Figure 1 shows the treatment effect estimates in study 1. The results are grouped into subfigures by substantive outcome area. The leftmost column in each subfigure shows the outcome variable being estimated. The next column indicates whether the outcome was measured immediately after the conversation or in the follow-up survey we conducted approximately 3 months afterward. The center of the plot shows the point estimate for the effect of the Perfect Day condition, with estimates surrounded by standard errors (thick lines) and 95% confidence intervals (thin lines). Last, the rightmost column shows the adjusted *P* values; as described in Materials and Methods, these are adjusted using the false discovery rate correction procedure from Anderson (*30*). All variables are oriented such that positive coefficients correspond with the expected direction of the effects.

Table S3 provides the precise numerical results and the categorization of each outcome (as, e.g., primary, secondary, etc.). Table S5A shows the raw means of the primary outcomes by condition.

#### Manipulation checks

Figure 1A shows the results on the manipulation check items. As the Perfect Day condition only differed from the Placebo condition by informing participants that they were talking with an outpartisan, the main manipulation check of interest was an item asking participants whether they thought that they just spoke to an outpartisan. Reassuringly, we found large effects on this item (*d* = 0.81, *P*_adjusted_ = 0.001), indicating that the manipulation was successful.

We also found that participants in both conditions were similarly likely to talk about their perfect day and similarly likely to talk about unrelated topics, suggesting that treatment is not confounded with following the assigned conversation topics. Although all participants were told to talk about their perfect day, participants in the Perfect Day condition were more likely to say they had talked about politics (*P*_adjusted_ = 0.012), but only 24% somewhat or strongly agreed that they did so.

#### Intergroup attitudes (e.g., affective polarization)

Figure 1B shows the estimated effects of the Perfect Day condition on intergroup attitudes (e.g., affective polarization). The first row in Fig. 1B shows the estimated effects on our primary outcome of interest and the most direct measure of affective polarization—warmth toward outparty voters. We find that assignment to the Perfect Day condition caused a sizable increase in warmth toward outparty voters (*d* = 0.34, *P* < 0.0001). In terms of thermometer degrees, this is an effect of 9 degrees, meaning that the conversations reduced the equivalent of over two decades of increase in affective polarization, as chronicled by (*1*). However, the next row shows that this large reduction in affective polarization completely evaporated in our 3-month follow-up survey (*d* = −0.02, *P*_adjusted_ = 1).

The second group of rows shows that the Perfect Day condition also dramatically increased meta-perceptions of respect: Participants in the treatment group were much more likely to think that outpartisans respected members of their own party (*d* = 0.45, *P*_adjusted_ = 0.001). The next row shows that this result does not persist in our 3-month follow-up, however.

We found no effect on a different secondary outcome—humanization of the outparty. This might be due to a ceiling effect, as participants across conditions already rated outpartisans a 78 out of 100, where 100 is the most humanized.

Last, we found no effect on warmth toward inparty voters—a tertiary outcome. This shows that the Perfect Day condition did not simply increase positive affect generally, and indicates that there are effects on affective polarization defined as outparty warmth minus inparty warmth, as we found effects on the former but not on the latter.

#### Outcomes relevant to democratic accountability

A notable feature of our study is that we test the often-hypothesized but rarely tested claim that interventions that reduce affective polarization, such as cross-partisan conversations, would improve outcomes relevant to democratic accountability. Figure 1C shows the estimated effect of the Perfect Day condition on the outcomes we measured relevant to democratic accountability. As described in Materials and Methods, these outcomes span a broad range, reflecting the broad range of negative impacts scholars have worried affective polarization might have for democracy—impacts many therefore hope that cross-partisan conversations might ameliorate.

The results in Fig. 1C, however, are consistently null. Across all nine outcomes, we find no evidence that assignment to the Perfect Day condition improved outcomes relevant to democratic accountability: We found no evidence that having a cross-partisan conversation caused individuals to be more likely to prioritize issue agreement over partisanship when selecting a candidate, to embrace policy views associated with the outparty, to support legislative bipartisanship, to avoid voting for copartisan candidates who violated democratic norms, to express openness to talk to outpartisans about politics, to say that it is important for outpartisans to discuss politics, or to seek out more level media coverage. One item in this area achieved statistical significance at conventional levels (warmth toward outparty politicians), but this estimate was not significant after adjusting for multiple comparisons (*P*_unadjusted_ = 0.039, *P*_adjusted_ = 0.377) and returned to baseline in the long-term follow-up. We also did not find effects on warmth toward inparty politicians in either immediately or long-term. Overall, then, despite the large decreases in affective polarization (i.e., large increases in warmth toward outpartisans) we found, we find no evidence that these increases had downstream effects for outcomes relevant to democratic accountability.

#### Potential mechanisms and moderators

Figure S2 reports results from our tests for potential mechanisms and moderators.

Regarding mechanisms, we found suggestive evidence that the Perfect Day condition caused participants to see the outparty as more similar, consistent with our theoretical reasoning. Although the point estimate was large, this result was marginally significant at conventional levels before adjusting for multiple comparisons and not after doing so (*d* = 0.18, *P*_unadjusted_ = 0.050, *P*_adjusted_ = 0.377). We do not perform a formal test for mediation because the sequential ignorability assumption would not be plausible in this context (*31*), but future research could seek to manipulate this mediator with additional conditions to more conclusively determine its role.

We also saw some directional evidence that the items from the receptiveness to opposing views index (*32*) moderated the effect of the Perfect Day condition, although this was not statistically significant. (In response to feedback, we conducted a post hoc test, which found similar effects on effects toward outparty voters for Democratic and Republican respondents; see fig. S7.)

#### Summary of study 1

In summary, study 1 found that cross-partisan conversations about their perfect day can dramatically reduce affective polarization and boost respect meta-perceptions (or the perception that the outparty respects members of the inparty). The reductions in affective polarization we found were substantively quite large, reversing approximately two decades worth of increases. However, we found that these effects did not persist in the long-term and decayed within 3 months. Moreover, consistent with our argument that the effects would be circumscribed within the political domain, we also found that, even immediately, the conversations did not appear to have positive downstream effects for a broad range of attitudes relevant to democratic accountability. These effects may have arisen because of increases in perceived similarity, although our evidence on the mechanism responsible was inconclusive. Study 1 left open several questions, including about the potentially conditional nature of these effects on the topic discussed, which we investigate in study 2.

### Study 2: Does encouraging participants to discuss areas of disagreement undermine the effects of cross-partisan conversation?

Study 2 included both conditions in study 1 (i.e., the Perfect Day and Placebo conditions) and two additional conditions: an Inparty Strengths condition in which we asked participants to discuss why they felt closer to their party and an Outparty Flaws condition in which we asked participants to discuss why they did not feel closer to the other party (see Table 1 for an overview of all the conditions and their differences). The exact prompts were “If you feel closer to the Democratic Party, what do you like about the Democratic Party? If you feel closer to the Republican Party, what do you like about the Republican Party?” (Inparty Strengths) and “If you feel closer to the Democratic Party, what do you not like about the Republican Party? If you feel closer to the Republican Party, what do you not like about the Democratic Party?” (Outparty Flaws).

Study 2 therefore explores whether encouraging participants to discuss areas where they disagree would undermine the salutary effects of cross-partisan conversation. It is by no means obvious that it would: There is more agreement between partisans than they expect (*13*), and so even discussions ostensibly focused on differences between partisan groups may surface more points of agreement between individuals than they expect. Many “depolarization” groups encourage partisans to explicitly discuss disagreements—e.g., discussing salient differences between groups (rather than, e.g., issues where there is substantial cross-partisan agreement) (see, e.g., livingroomconversations.org, braverangels.org, and https://openmindplatform.org/). However, inspired by research regarding the potential pitfalls of discussing differences between groups (*11*), we theorize that encouraging participants to discuss areas of disagreement might undermine the potential for cross-partisan conversations to reduce affective polarization.

This reasoning led us to include the Inparty Strengths condition in study 2, in which we ask participants to each discuss why they support their party. The contrast between this condition and the Perfect Day condition was intended to manipulate the extent to which the conversation prompts encouraged participants to discuss areas of agreement and similarities instead of disagreements and dissimilarities. Whereas prior research shows that the Perfect Day prompt leads people to agree and notice areas of similarity (*24*) (and we find some evidence in study 1 consistent with this), the one and only thing we knew differed between outpartisans was their partisan preferences, and so we selected discussions of their partisan preferences to maximize the chances of discussing a disagreement. While previous literature has already shown that discussions of political similarities can reduce affective polarization (*15*), we wanted to test whether instead focusing on disagreement (as practitioners often attempt to do) might be counterproductive.

Two aspects of our choice of topic for the Inparty Strengths condition bear further comment. First, our choice of topic means that two things changed between the Perfect Day and Inparty Strengths conditions: whether the conversations encourage disagreement instead of agreement and whether they encouraged the participants to dwell on partisanship. However, prior research by (*15*) indicates that encouraging participants to dwell on partisanship in a conversation does not prevent reductions of affective polarization, so we concluded that the mere introduction of partisanship as a topic alone was unlikely to represent an alternative explanation for our findings. Second, we encouraged participants to discuss their partisan preferences instead of a specific issue. We did so because rank-and-file outpartisans often actually agree with each other on individual issues, so it is not guaranteed or even always probable that conversations about individual issues would dwell on areas of disagreement; they may instead surface areas of unexpected agreement. Since our intent was to vary encouragement to discuss areas of agreement or disagreement as much as possible, we therefore prioritized encouraging participants to discuss the one area we knew our participants disagreed on: their partisan preferences. Future research should continue to investigate the impacts of cross-partisan conversations that focus on a specific issue, however.

The Outparty Strength condition was inspired by the fact that practitioners often are interested in having outpartisans discuss intergroup differences. We thought that the Outparty Strength condition might allow participants to discuss differences while actually surfacing less disagreement. This is why the Outparty Flaws asked participants to discuss why they do not identify with the other party. In light of the fact that partisanship is characterized more by negative feelings toward the outparty than positive feelings toward one’s inparty [for review, see (*6*)], we thought that partisans might actually agree with some criticisms of their party made by their conversation partners, surfacing unexpected areas of agreement between them.

Another difference between studies 1 and 2 is that study 2’s participants were recruited with Facebook ads, providing a more representative sample. Study 2 materials and methods were largely similar to study 1 materials and methods, with minor differences described in Materials and Methods.

#### Sample characteristics

*N* = 2541 participants completed the screener. *N* = 607 began the conversation survey and then clicked the page containing a link to the conversation. Last, *N* = 338 participants were able to successfully begin a conversation. Table S2 shows the demographics of the sample at each stage. As determined by the audio recordings, the Placebo conversations lasted a median of 13.4 min, the Perfect Day conversations lasted a median of 13.6 min, the Inparty Strengths conversations lasted a median of 13.7 min, and the Outparty Flaws conversations lasted a median of 17.9 min.

Reversing the pattern found in study 1, participants in the Placebo condition were directionally more likely to have a conversation compared to those in the Perfect Day condition (*P* = 0.547), and participants in the Outparty Flaws condition were less likely to have a conversation compared to the Placebo condition (*P* = 0.034), but there was no evidence of difference in attrition by condition overall [*F*(603) = 1.63, *P* = 0.181]. There was also no evidence of differential attrition by covariates, as assessed by a joint hypothesis test on the interactions between covariates and treatment assignment on a regression predicting attrition (*P* = 0.766) (*29*). Last, table S2 shows that baseline covariates were balanced among the experimental groups.

#### Estimated treatment effects

**Fig. 2.**
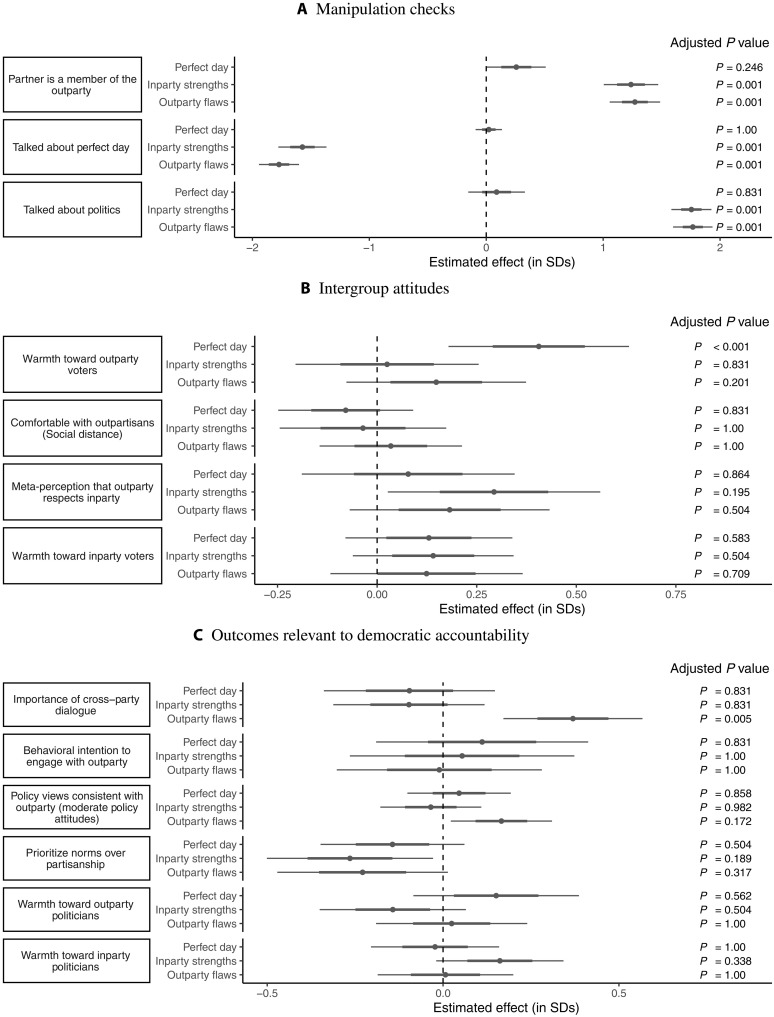
Study 2 results. Notes: Points show the estimated effects of each of the conditions (shown on the *y* axis labels) relative to the Placebo condition in study 2. Standard errors (thick lines) and 95% confidence intervals (thin lines) surround the point estimates. Adjusted *P* values are adjusted across all nonprimary outcomes using the procedure outlined in (*30*). As described in the text, *P* values for primary outcomes are not adjusted. (**A**) Manipulation checks. (**B**) Intergroup attitudes. (**C**) Outcomes relevant to democratic accountability.

Figure 2 shows the estimated treatment effects in study 2. The results are grouped into subfigures by substantive outcome area. The leftmost column in each subfigure shows the outcome variable being estimated. The next column indicates which condition’s effects are being estimated (always relative to the Placebo condition). The center of the plot shows the point estimate for the effect of each condition relative to the placebo group, with estimates surrounded by standard errors (thick lines) and 95% confidence intervals (thin lines). Last, the rightmost column shows the adjusted *P* values; as described in Materials and Methods, these are adjusted using Anderson’s 2008 False Discovery Rate correction procedure. Except for the manipulation checks (where predictions go in opposite directions for different conditions), all variables are oriented such that positive coefficients correspond with the expected direction of the effects.

Table S4 provides the precise numerical results and categorizes the outcomes by whether they are primary, secondary, etc. Table S5B shows the raw means of the primary outcomes by condition.

##### Manipulation checks

First, Fig. 2A shows the effects of each condition on the manipulation checks. The results indicate that the manipulations were broadly successful. As expected, participants in the Inparty Strengths and Outparty Flaws conditions were aware that they were talking with an outpartisan. These effects dwarfed that of the Perfect Day condition, which, although directionally in the right direction, did not reach statistical significance after adjusting for multiple comparisons (*P*_unadjusted_ = 0.049, *P*_adjusted_ = 0.246). In addition, participants appeared to comply with their assigned conversation topics: Those in the Perfect Day condition were similarly likely as those in the Placebo condition to talk about their perfect day (the topic assigned to both conditions), whereas those in the Inparty Strengths and Outparty Flaws conditions were much less likely to say so. By contrast, those in the Placebo and Perfect Day conditions were similarly likely to say that they talked about politics, but participants in the Inparty Strengths and Outparty Flaws conditions were much more likely to say that they talked about politics.

##### Intergroup attitudes

Next, Fig. 2B shows the results on intergroup attitudes. Similar to study 1, we found large, positive effects of the Perfect Day condition versus Placebo on warmth toward the outparty (*d* = 0.41, *P* < 0.001). However, neither of the conditions prompting participants to talk about their partisan preferences had statistically significant effects on outparty warmth.

Moreover, we can statistically distinguish the effects of the Perfect Day condition from the political conditions. Figure S5 shows that while mean warmth toward outparty voters in the political conditions does not differ from Placebo (*P*_adjusted_ = 0.40), it is significantly lower than in the Perfect Day treatment group (*P*_adjusted_ = 0.015). This indicates that encouraging discussion of disagreements reduced the effects of the conversations on affective polarization, possibly to zero.

No other measure of intergroup attitudes reached significance after correcting for multiple hypothesis testing, including the meta-perception that the outparty respects one’s own party (*d* = 0.08, *P*_unadjusted_ = 0.573, *P*_adjusted_ = 0.994), although the point estimate for this item was also not significantly different than the significant effects we found in study 1.

##### Outcomes relevant to democratic accountability

Similar to study 1, we found consistently null results on outcomes relevant to democratic accountability, with one exception. We found null results on behavioral intentions to engage with the outparty, on holding policy views consistent with the outparty, on prioritizing norms over partisanship when voting, and on views toward outparty politicians.

Surprisingly, there were limited exceptions to this pattern of null results for the Outparty Flaws condition. First, this condition increased perceptions regarding the importance of cross-partisan dialogue, a secondary outcome. This result remains highly significant even after correcting for multiple comparisons (*d* = 0.37, *P*_unadjusted_ < 0.001, *P*_adjusted_ = 0.005). We also found suggestive evidence that this condition may have reduced attitudinal polarization by causing voters to express policy views more consistent with the outparty (i.e., that were more moderate); this result invites replication as it was not significant after multiple comparison adjustments (*d* = 0.17, *P*_unadjusted_ = 0.03, *P*_adjusted_ = 0.17). The point estimate on the effect of the Outparty Flaws condition on outparty warmth was also positive, although far from significance (*d* = 0.15, *P* = 0.201). Although preliminary, these counterintuitive results are consistent with our motivations for including the Outparty Flaws condition: Respondents may be more open to discussing pitfalls of the political parties than their virtues [see also (*33*)]. We return to this in Discussion.

##### Mechanisms

Figure S4A shows our findings for potential mechanisms and moderators. We generally find no evidence for any of our posited mechanisms: anxiety during the conversation, warmth toward one’s conversation partner, or feeling listened to by one’s partner. This suggests that the potential mechanism we identified in study 1, perceived similarity, may be most worthy of future research. One exception to the pattern of null results on mechanisms in study 2 is that we found evidence of a negative effect of the Inparty Strengths condition on feeling listened to in the conversation. This result survives a multiple comparison adjustment (*d* = −0.67, *P*_unadjusted_ < 0.001, *P*_adjusted_ = 0.004). This supports our argument about the potential disadvantages of encouraging participants to discuss areas of disagreement.

##### Moderation

Figure S4B finds no significant evidence of moderation across any of the preregistered moderators, except for one marginally significant negative interaction between extroversion and the Inparty Strengths condition, although the statistical power of these tests is limited.

#### Summary of study 2

Study 2 extended study 1’s results in a more representative sample and with two new conditions that encouraged discussions of disagreements, which enabled us to investigate the extent to which the topic of conversation influences the effect of cross-partisan conversation on affective polarization. Overall, study 1 replicated the promise of discussions across partisan lines, finding that such conversations dramatically increased warmth toward outpartisans. On the other hand, supporting our argument about how the effects of cross-partisan conversation are conditional on conversation topic (in particular, whether they encourage discussion of areas of disagreement), conversations encouraging participants to discuss areas of disagreement had no effects, a difference we can statistically distinguish from the Perfect Day conversations. Again, supporting our argument regarding the circumscribed effects of these conversations, we also again found no downstream consequences of this increased warmth for outcomes relevant to democratic accountability.

One potentially surprising pattern in the results of study 2 is that the Outparty Flaws condition appeared more promising than the Inparty Strengths conditions across several metrics (see also fig. S6). In particular, we found that those in the Outparty Flaws condition spent more time talking to their partner, were more likely to say that cross-partisan conversations were important, and may have even expressed less polarized policy attitudes and greater warmth toward the outparty (the latter two which were not significant). We also found evidence that, by contrast, those in the Inparty Strengths condition felt less listened to. Although the estimated effect of the Outparty Flaws condition on warmth toward outpartisans was far from statistically significant (*d* = 0.15, *P* = 0.20), this constellation of promising results suggests that future research into this approach is warranted.

## DISCUSSION

As concern about affective polarization has grown among scholars, activists, and organizations, so has excitement about the potential of cross-partisan conversations to reduce it. Accordingly, practitioners and scholars have proposed a variety of interventions—ranging from national service, to online apps, to “bridging” workshops—intended, among other things, to foster face-to-face conversation between outpartisans. However, the effects of such conversations remain theoretically ambiguous and have been subject to relatively little prior research. We argued that cross-partisan conversations have potential to reduce partisan animus, especially if they are about a topic that encourages discussions of areas of agreement. However, we also argued that these effects would be conditional on topic, diminishing if the conversations encourage participants to discuss areas of disagreement. We also argued that the effects of such one-shot interactions are likely to not persist in the long term and be circumscribed to intergroup attitudes, not extending to outcomes relevant to democratic accountability. We present two unique experiments supporting this argument in which we matched outpartisans to have face-to-face video conversations about randomly assigned topics, relying on custom software that we developed for this purpose.

Our results suggest several implications for efforts to reduce affective polarization and for research on intergroup attitudes more generally. First, our findings both confirm the promise of and indicate limits of cross-partisan conversations: Such conversations appear able to temporarily reduce partisan animus toward outpartisans but may not lastingly shift it in the long-run absent complementary manipulations.

Second, although partisan groups and other groups (e.g., racial groups) are different, our findings suggest implications for the literature on intergroup contact. In light of a recent review finding that very few studies of intergroup contact both feature random assignment and track long-run effects (*10*), our finding of immediate large effects followed by complete long-run decay is notable. Furthermore, most intergroup contact research has focused on interactions between majority and minority groups and not between members of the political parties [see (*12*)]. Our finding that contact between Democrats and Republicans can reduce partisan animus is notable as this contact may have met only one of the four conditions Allport (*18*) theorized would be necessary for contact to improve intergroup attitudes: Participants may have felt equal status as there was no difference in how respected participants said they felt (see fig. S4), but we did nothing to encourage participants to cooperate, have a shared goal, or feel institutional support. However, in light of other studies that have found that brief conversations can durably improve intergroup attitudes when one conversation partner is trained in specific techniques [e.g., (*17*)], our results point to the need for further study of the necessary conditions for conversations to improve intergroup attitudes in a durable rather than evanescent manner.

Third, our findings suggest that what is talked about is as important as whether there is conversation at all. Our results suggest that discussing a topic that fosters a discussion of shared views or experiences, rather than discussing areas of disagreement, may prove a more effective way of reducing affective polarization. This finding complements those from other studies that find that highlighting unexpected interpartisan similarities can reduce multiple forms of polarization [e.g., (*13*, *14*, *16*)] as well as studies that find that having partisans dwell on issues where there is likely to be agreement across parties can reduce polarization [e.g., (*15*)]. Along these lines, our evidence suggested that conversations about people’s perfect day may have increased perceived similarity. Consistent with field research in other domains (*11*), our results suggest that explicit discussions of areas of disagreement may undermine the salutary effects of intergroup contact in the political domain. With this said, we cannot rule out the possibility that the null effects we observed in the Inparty Strengths and Outparty Flaws conditions could have been driven by the prompts leading participants to discuss their partisan identities, as opposed to disagreement. Although results in (*15*) (finding that discussions centering around areas of agreement between partisan identities did reduce affective polarization) can help allay this concern, a study that instructs participants to explicitly discuss identities could help rule out the possibility that the null effects were due to identities being the focus of the conversational prompts.

Fourth, our findings question many scholars’ and practitioners’ assumption that interventions that reduce affective polarization will have salutary consequences for democratic accountability; we found no such effects, in line with other recent findings on the effects of other interventions (*25*). The most likely way in which reductions in affective polarization might lead to improving democratic accountability attitudes would be for cross-partisan conversations to improve attitudes toward outparty voters and, in turn, improve attitudes toward outparty elites, which might then improve various more general attitudes (e.g., support for legislative bipartisanship). In both studies 1 and 2, although the Perfect Day condition increased warmth toward outparty voters, there was no effect on warmth toward outparty politicians, suggesting a broken link in this potential causal chain. This is in line with Druckman *et al.*’s (*34*) finding that attitudes towards voters and elites are distinct. Of course, our studies only allow us to rule out the hypothesis that brief contact with an outpartisan would change attitudes toward democratic accountability. Our results do not speak to whether or not more sustained engagement or interventions with greater training and facilitation, such as which occurred in the America in One Room experiment (*35*), might lead to changes in political attitudes or behaviors. Future research may also wish to examine more sustained engagement (e.g., repeated interaction with outpartisans) as well as conversation prompts, which more explicitly elicit discussion of political elites or democratic norms.

Fifth, though, for practitioners who see normative value in having partisans discuss their differences, our findings regarding the impacts of having partisans discuss what they dislike about the other party (i.e., the Outparty Flaws condition) were surprisingly promising. We thought that this condition might paradoxically lead to less disagreement—as most individuals do not like their party particularly strongly. Consistent with this, we found that participants spent longer on these conversations than the other topics and that they later were more likely to rate cross-partisan dialogue as important after having them. These findings resonate with research on the benefits of exposure to cross-cutting views (*36*). In a qualitative review of some of the conversation audio recordings, we found that many of the Outparty Flaws conversations featured individuals agreeing with the criticisms or shortcomings that their conversation partner mentioned about their own party, which may have disconfirmed stereotypes about the other party’s extreme views. In future research, we plan to further investigate this potential mechanism.

Last, our results point to the need for further experimentation on the necessary conditions for conversations to improve intergroup attitudes generally. Other studies have found that brief conversations can durably improve intergroup attitudes when one conversation partner is trained in specific conversational techniques [e.g., (*17*)]. For example, might approaches for making others feel listened to [e.g., (*37*)] or being more receptive [e.g., (*38*, *39*)] make the conversations more impactful or memorable? Might different approaches to fostering deliberation [e.g., (*40*)] produce longer-lasting effects? Or might more sustained engagement or the presence of a skilled moderator do so (*35*)? Such questions about whether it is possible to instruct laypeople to have more productive cross-partisan conversations than they are by default remain a question for future research. Relatedly, research will be necessary to understand how to scale these conversations. Although a number of nonprofits have already had success rolling out interventions that include cross-partisan conversations, research is only beginning to understand how best to scale such interventions.

There are several limitations to our work. Many of these limitations arise because of the constraints imposed by studying these conversations experimentally—which not only brings important methodological advantages but also may lead the conversations we studied to not resemble real interactions for many people or the outcomes of greatest interest. First, although face-to-face online conversations are important to study in their own right, our results might have differed if we had studied other mediums of conversations—such as phone conversations, in-person conversations, etc. Second, we chose to study conversations between outpartisan strangers in which we instructed them to talk about a specific topic, given that this is the model adopted by most nonprofits attempting to reduce affective polarization. However, our findings may not generalize to naturally occurring conversations between friends, co-workers, or relatives, and future research should explore this. Such research would aid scalability. Third, future studies could benefit from more unobtrusive measures, such as behavioral measures, and efforts to further conceal the purpose of the studies to reduce the potential for demand to influence the results. With that said, the null results of the Inparty Strengths and Outparty Flaws conditions in study 2 suggest that our positive results were not due to demand, although it is possible that these null results could have arisen because the negative experience of disagreeing counteracted the effects of demand. Fourth, although one of our samples relied on recruiting members of the general public over Facebook, future studies should replicate our findings in more representative samples. In particular, since our findings are limited to people interested in having a video conversation with a stranger, future studies should attempt to incentivize those who are less interested in such conversations, who may differ from our sample on unobservable traits. Fifth, our studies were well powered to detect reasonably small effects (the standard errors on our primary hypotheses tests were equal or smaller to 0.10 standard deviations), in part because including pretreatment covariates in our preregistered specifications reduced the standard error of our estimates (*29*). (Specifically, on our primary hypothesis test for effects on outparty warmth, study 1’s statistical power was equivalently well powered to a study without pretreatment covariates with a sample size of *N* = 1264, and study 2’s statistical power was a equivalently well powered to a study without pretreatment covariates with a sample size of *N* = 690.) However, future studies may need larger sample size to any detect effects too small for our studies to uncover.

Together, however, our results do suggest several important conclusions. First, we find that merely having a face-to-face conversation with an outpartisan reduces affective polarization. We also find that what is talked about matters just as much as whether the conversation occurs at all: The effects shrink substantially, potentially to zero, when the conversations prompt participants to discuss areas of disagreement. Moreover, the effects of the cross-partisan conversations on affective polarization we found did not persist long-term, as they decayed to effectively 0 after 3 months; were circumscribed to interpersonal attitudes; and largely did not extend to democratic accountability. On a theoretical level, these findings lend further credibility to the promise of intergroup contact, even in cases when the conditions thought to be necessary for it to improve intergroup attitudes (e.g., cooperation) are not met. But for both scholars and practitioners, our findings also suggest that such conversations are not enough alone to result in the durable changes in attitudes toward outpartisans and toward democracy that many hope for.

## MATERIALS AND METHODS

The studies reported herein were approved by the Committee for the Protection of Human Subjects at University of California (UC) Berkeley and at Stanford University.

### Study 1 materials and methods

Study 1 took place in 2021. Figure S1 summarizes the methods and exclusions in study 1 that we describe below.

### Screener survey

We began by recruiting participants to “qualify for a 30 minute survey with a video call” on Prolific and Amazon Mechanical Turk. In this screener survey, we asked participants their party identification, their interest and availability to do a study involving a video conversation at specific dates on times, three attention checks, and for them to conduct a test of whether their computer was compatible with our software to have video conversations. We did not tell participants we wanted them to talk to an outpartisan specifically or to talk about politics. We then screened out participants based on several criteria (e.g., suspicious IP addresses) and recruited those who qualified to the full study via email, as described in more detail in the Supplementary Materials (part B.1).

Before launching the main conversation survey, we filed a preanalysis plan. The preanalysis plan is available at https://aspredicted.org/blind.php?x=ud7a62. After cleaning the data from the conversation survey but before examining study 1’s results, we filed an amended analysis plan that clarified several ambiguities in our original plan. The amended analysis plan is available at https://osf.io/q3hkx/?view_only=6575d00b686c418eb5591b66ca3ad628.

### Conversation survey

On the date and time assigned to each participant, we sent them an invitation to immediately begin the video conversation survey. Some participants received a message asking them to come back to the survey later if there were too many members of their party already waiting to be paired. After consenting to the study, in which participants were invited to “participate in a research study between strangers” and that this would involve talking “over video about an assigned topic,” we asked participants several baseline measures and then told them that they would be soon entering a video call platform, AllSides, where they were to have a 10-min conversation with someone also taking the survey.

The survey then displayed a link participants were instructed to click to join the video call. When participants clicked the link, they were directed to a custom landing page we created. The random assignment and partner matching took place in real time in the moments after participants clicked this link and before the landing page loaded. To assign participants to a condition, our software first checked whether any other participants of the other party were available (i.e., were still waiting to be paired). If there were no available outpartisans, the random assignment to condition then took place: The software randomly assigned participants to either the Placebo or Perfect Day conditions. If there were available outpartisans, participants were matched with the outpartisan survey participant who had been waiting the longest for a conversation partner and inherited this participant’s random assignment. (To account for this procedure, our standard errors are clustered by dyad.)

After this matching and assignment process completed (essentially instantaneously), participants arrived on a custom landing page that told them the topic of the conversation, which in all cases in study 1 was “What does the ‘perfect day’ look like to you?” (*24*). The website also told all participants “You will be matched with someone.” If participants had been randomly assigned to the Perfect Day condition, the sentence went on to say “who feels closer to the [OUTPARTY] party. (You told us you feel closer to the [INPARTY] party).” Participants in the Placebo condition were not told this. The experimental manipulation in study 1 is therefore whether participants were aware that they were talking with an outpartisan, given that a conversation began. This allowed us to hold constant the ability of participants to be matched with an outpartisan given the specific time at which they appeared and for their computer software to be working. In Results, we show that this manipulation had a very large effect on whether participants believed that they were talking with an outpartisan.

After this page displayed for 12 s, participants were redirected to a video chat room on the AllSides Connect website (allsidesconnect.com), a video platform that graciously agreed to allow us to use their platform for our studies. (The video was automatically turned on for participants. Unfortunately, we do not have the data on whether or not a participant had their video on.) This video chat room was specific to them and their partner. The instructions on the screen in the room again told participants the topic of the conversation and, if the participants were in the Perfect Day condition, reminded them that one participant felt closer to the Democratic party and the other the Republican party. The site also asked participants to wait up to 5 min for a partner to arrive (as some participants were the first instead of the second in their pair). All conversations were between two participants only, one Democrat and one Republican. Participants received no further instructions about how to have the conversation as we were interested in the effect of conversations between untrained laypeople for this initial investigation, rather than seeking to evaluate any one particular set of further instructions.

After having the conversation, participants were instructed to continue with the survey (which could only advance after 5 min had elapsed). After indicating whether or not the conversation happened, participants then completed a series of dependent measures, detailed below. At the end, participants were debriefed and thanked.

A median of 3 months (to be exact, 84 days) after the surveys took place, we contacted participants asking them to complete a follow-up survey. We chose approximately 3 months given previous findings that the effects of persuasive conversational interventions can last at least this long [e.g., (*17*, *41*)]. The follow-up survey contained a subset of the outcome variables on the original conversation survey (asked in the exact same manner), as well as an additional measure, “Importance of Cross-Party Dialogue” (see below). Although the follow-up survey invitation told participants that they qualified because they had taken a previous study with us, the invitation and survey made no reference to the specific nature of the study.

### Outcome variables and other measures

We asked our outcome variables on the conversation survey after the conversation had ended. We also asked a subset of these variables in the 3-month follow-up survey.

#### Manipulation checks

We first asked a series of manipulation checks about what happened in the conversations, as well as whether participants thought that their partner was an outpartisan. These were our main manipulation checks, as the Perfect Day condition was identical to the Placebo condition except for informing participants that they were speaking to an outpartisan.

#### Intergroup attitudes

We asked several measures to tap intergroup attitudes. As we preregistered, our first primary outcome was our primary measure of affective polarization, outpartisan affect: a feeling thermometer measuring affect toward outpartisan voters (*26*). (Affective polarization is often defined as the difference in affect toward the outparty minus the inparty. We focus on the warmth toward outpartisans component for simplicity and because the conversations did not target reducing feelings of warmth toward one’s own inparty; in Results, we verify that none of the conversations had significant effects on warmth toward one’s own inparty.)

We also asked a second primary outcome measure about respect meta-perceptions: “In general, how respectful do you think people who vote for [outparty]s are of people who vote for [inparty]s?” from “not at all respectful” to “very respectful.” We developed this measure in light of recent research suggesting the importance of meta-perception in explaining, and reducing, affective polarization (*42*).

Last, we also asked two questions we preregistered as secondary outcomes. First, we measured humanization of the outparty, measured using the question: “People can vary in how human-like they seem. Some people seem highly evolved whereas others seem no different from lower animals,” and then asking participants to move a sliding scale corresponding to the “ascent of man” measure adapted from (*43*) [for prior use in a partisan context, see (*44*)]. Second, we measured warmth toward inparty voters (which we asked to ensure that inparty affect was not also increasing in tandem; we measured this using a feeling thermometer).

#### Outcomes relevant to democratic accountability

One of the notable features of our study is that we examine the potential for cross-partisan conversations to have positive downstream effects for outcomes relevant to democratic accountability, as many scholars and organizations hope. There are no widely agreed upon measures of this concept as such impacts have rarely been studied, and so we measure a wide variety of outcomes in this area.

First, our third and final primary outcome assesses choosing an outpartisan candidate aligned on issues. To measure this, we displayed a table to participants that showed two hypothetical candidates for office, the candidates’ parties, and the candidates’ positions on two issues (gun rights and abortion). The inpartisan candidate had issue positions opposite the participant, which had been measured pretreatment, and the outpartisan candidate had the same issue positions as the participant. Inspired by the worry that affective polarization would “weaken...willingness to punish one’s own party’s politicians” [(*5*), p. 50] for taking incongruent positions, we asked participants which candidates they would vote for, and measured whether participants assigned to the Perfect Day condition would be more likely to select the candidate aligned with their issue views instead of their party. In Little *et al.*’s (*45*) framework, this measures both divergence and desensitization. All our remaining outcomes in this area were preregistered as secondary outcomes unless otherwise noted.

Second, to see whether conversations might lead to less polarized policy views, we measured participants’ views on several issues and computed an index of how often their policy attitudes were consistent with the outparty rather than inparty (e.g., a Republican having a liberal view on abortion). In the literature on affective polarization, this is sometimes referred to as attitudinal polarization; this is relevant to democratic accountability because it speaks to “partisans’ willingness to conform to their party’s policy positions” rather than hold their party accountable for these positions [(*6*), p. 142].

Third, to test the hypothesis that reducing affective polarization with cross-partisan conversations might increase support for legislative bipartisanship (*7*), we adapt a vignette from Harbridge and Malhotra [(*46*), study 2]. The vignette tells participants about an actual Member of Congress of their party, and it is randomly assigned whether they learn about the year of votes when this copartisan member cast party-line votes or often cast votes with the outparty (i.e., in a bipartisan manner). We test whether the Perfect Day treatment causes participants to be additionally approving of the bipartisan-voting relative to the party-line–voting Member of Congress.

Fourth, to test the hypothesis that the conversations might make participants more willing to vote for outpartisan politicians to avoid voting for copartisan politicians who violated democratic norms, we adapt a series of items from Voelkel and colleagues (see https://osf.io/7evmp/). These items ask how likely participants would be to vote for copartisan candidates who commit various norm violations (e.g., would ignore unfavorable court rulings by outparty judges). We form an additive index of these items (“Prioritize Norms over Partisanship”).

Fifth, we asked participants how likely they would be to talk to an outpartisan neighbor about politics, and sixth, we asked about the importance of cross-partisan dialogue, in particular, asking individuals three items about how important they thought it was for members of their party to engage in conversation with outparty members (e.g., “Talk to people who vote for [OUTPARTY] about politics.”). We categorize these outcomes as relevant to democratic accountability because conversations between citizens are thought to help limit elite influence and organize collective action [e.g., (*47*)]. To measure the importance of cross-partisan dialogue, we created an additive index of multiple items that only appeared in the follow-up survey; this measure was not preregistered. Seventh, we asked participants whether they wanted to subscribe to a newsletter that provides bipartisan news to test the hypothesis that the conversations might reduce selective exposure to congenial information.

Eighth and (finally) ninth, we asked about warmth toward outpartisan and inpartisan politicians on a feeling thermometer. These were registered as tertiary outcomes. We classify them as relevant to democratic accountability because they speak to the likelihood of engaging in party-line voting. They are also a mechanism by which many of the other potential effects on outcome relevant to democratic accountability might be expected to manifest.

#### Mechanisms

Study 1 asked about two potential mechanisms. The first, perceived similarity, was measured with the question: “How similar is the typical [OUTPARY MEMBER] to you?” from “not at all similar” to “extremely similar.” We hypothesized that knowingly talking to an outpartisan about the perfect day might increase perceived similarity by revealing commonalities between the participant and their conversation partner, and thereby decrease partisan animus.

Second, we asked whether one’s partner was perceived as engaged in deep listening with the questions: “My partner shared a story about their perspective” and “My partner asked me about what I thought.” In light of work suggesting that perspective-getting might improve intergroup attitudes (*41*), we theorized that the extent to which partisans perceived their partner listening might decrease affective polarization.

#### Moderators

As we preregistered, we examine moderation of the treatment effects across three moderators that we asked about before treatment: frequency of having conversations with outparty; an index of political knowledge (*48*); and two items from an index of receptiveness to opposing views (*32*).

### Analytical approach

As we preregistered, we used linear regressions to estimate effects on all variables, regressing each outcome on an indicator for treatment assignment (with Placebo as the baseline) and pretreatment covariates to increase precision (*29*). [The covariates are the baseline value of the dependent variable being estimated (where available)—age, gender, education, race and ethnicity, party identification, and party ideology. For the test of support for bipartisanship, the coefficient of interest is the interaction between the bipartisanship treatment in the vignette and the conversation condition; for all other hypotheses, the coefficient of interest is the coefficient on the treatment indicator. For our tests of moderation, we included interactions between our posited moderators and the treatment indicator.] Because of these pretreatment covariates, the effective sample size and statistical power of our study is considerably larger. All standard errors are clustered at the dyad level given the random assignment procedure.

Because of the number of dependent variables we examine, we preregistered an approach for adjusting our *P* values for multiple comparisons using the approach outlined in (*30*). In particular, we do not adjust the *P* values for our three primary outcomes. We adjust all of the *P* values for the remaining outcomes, including our secondary outcomes, tertiary outcomes, mechanisms, manipulation checks, and moderation tests. (This is a more conservative approach than we preregistered, which was to adjust by domain. We made this decision before looking at results.) We also separately adjust the *P* values for all of the hypotheses tested on our 3-month follow-up survey. Under Anderson’s (2008) procedure, the resulting adjusted *P* values can be interpreted as controlling the false discovery rate; for example, using the traditional threshold of 0.05, 5% of adjusted *P* values under this threshold are expected to be false positives.

We rescaled all dependent variables to SD one, so all estimates we report are in SDs. The estimates are therefore equivalent to the Cohen’s *d* effect size.

#### Exclusions

Because of the unpredictability of how many participants of each party would show up for the study at any given time and the need to match participants with outpartisans, a number of participants in the conversation survey were not ultimately matched with an outpartisan because none arrived in time. We exclude observations from our analyses where participants never clicked the link to be randomly assigned and join the conversation platform, were never matched with a conversation partner, or, due to technical problems, were unable to begin a conversation. We include all cases where a conversation began, and so our estimates are complier average causal effects [i.e., “CACE”; (*29*)]. We identify these cases using questions we asked participants immediately after the conversation about whether a conversation took place and, if not, why not, as well as data from the AllSides platform on whether two people joined the AllSides room they were assigned. In cases where we received conflicting information from participants or from the AllSides platform about whether a conversation began, we resolved discrepancies by manually reviewing a recording of the audio from their AllSides conversation room, which we captured with participants’ prior consent. (Recordings were not captured for a small number of participants due to a coding error, so we rely solely on their self-reports for these cases.) We determined all of these exclusion criteria and which individual recordings we would and would not exclude blind to treatment assignment and before examining study 1’s results. Figure S1 provides a graphical overview of the recruitment process and the exclusion criteria.

### Study 2 materials and methods

We recruited participants to study 2 in 2021. Figure S3 summarizes the methods and exclusions in the first half of study 2; as described below, we updated the technology setup (mirroring study 1) halfway through study 2, and so fig. S1 describes the second half of study 2.

### Screener

Participants were first recruited via Facebook ads to a screener survey. In this screener, we asked participants a suite of measures to assess political beliefs (party identification, political ideology, and political knowledge), baseline scores for several outcome measures (see below), moderators (see below), demographics (age, education, gender, race and ethnicity, residency, and citizenship), their interest and availability to do a video study, system compatibility, and their contact information. We screened out participants based on several criteria (e.g., suspicious IP addresses) and then invited those who qualified to the main study, as detailed in the Supplementary Materials (part B.2). Before beginning the conversation survey, we filed a preregistration. Our preregistration is available at https://aspredicted.org/blind.php?x=43v3vh.

### Conversation survey

The conversation survey proceeded in a similar manner to study 1. After consenting, participants then were told that they would be soon entering a video call platform, AllSides, where they were to have a 10-min conversation with someone also taking the survey.

#### Experimental conditions and random assignment

Table 1 gives an overview of the experimental conditions in study 2 and how they compare to the conditions in study 1. As in study 1, study 2 had the Placebo condition, where participants were matched with an outpartisan to talk about their perfect day, but were not told that they were matched with an outpartisan, and a Perfect Day condition, where they were informed that they were discussing their perfect day with an outpartisan. We also added two new conditions to study 2. First, in the Inparty Strengths condition, we instructed participants to talk with each other about what they like about the party they feel closer to. (The exact prompt was “If you feel closer to the Democratic Party, what do you like about the Democratic Party? If you feel closer to the Republican Party, what do you like about the Republican Party?”) Second, in the Outparty Flaws condition, participants discussed what they did not like about the other party. (The exact prompt was “If you feel closer to the Democratic Party, what do you not like about the Republican Party? If you feel closer to the Republican Party, what do you not like about the Democratic Party?”)

We used the same matching procedures as in study 1. However, before we deployed updates to the software halfway through study 2, if participants were in any of the treatment conditions, we told them that they would be having a conversation with an outparty member (though they were not told about what) in the survey itself before them clicking the link (instead of using a landing page); the remainder of the participants were told the party of the partner (if in the treatment condition), and the conversation prompt, on the landing page after clicking on the link (as happened in study 1).

After having the conversation or waiting for at least 5 min for a partner to arrive, participants were told to continue with the survey (they could only advance after 5 min). After indicating whether or not the conversation happened, participants then completed a series of measures, detailed below. At the end, participants were debriefed and thanked.

### Outcome variables and other measures

Study 2 included several outcome variables also asked in study 1 in an identical manner; for brevity, we only list them here: perception that partner is a member of the outparty, whether they talked about the perfect day, whether they talked about politics (manipulation checks); warmth toward outparty voters, meta-perception that outparty respects inparty, warmth toward inparty voters (intergroup attitudes); importance of cross-party dialogue, policy views consistent with outparty, prioritize norms over partisanship, warmth toward outparty politicians, and warmth toward inparty politicians (outcomes relevant to democratic accountability).

Study 2 included three outcome measures not included in study 1. The first two outcomes were in the intergroup attitudes category. First, we formed an index of three measures of comfort with outpartisans (social distance): how upset (not at all upset to extremely upset) participants would feel if their son or daughter married a member of the outparty and how comfortable they were of having outparty friends and neighbors (not at all comfortable to extremely comfortable) (*1*).

Second, we asked participants whether they felt respected by their partner, i.e., “My partner respected me,” “My partner respected my feelings,” and “My partner did not respect my opinions” (reverse-scored) from “strongly disagree” to “strongly agree.” We created an additive index from the three items.

We categorized the third new outcome as relevant to democratic accountability, whether participants expressed a behavioral intention to engage with outparty. We measured this with three items: Participants were asked to indicate the extent to which they would be interested in having another conversation with a member of the outparty, if they would learn from such a conversation, and of the participants who indicated that they had a conversation, if they would be interested in meeting up with their partner again. We formed an index with all three items.

#### Moderators

Study 2 also contained several additional preregistered moderators measured before the conversation: extroversion and openness to experience (*49*); frequency of previous political conversation with the outparty (measured as in study 1); self-monitoring (*50*); and a political knowledge index (*48*).

#### Mechanism measures

After the conversations, study 2 also collected three preregistered potential mediators: anxiety during the conversation [an additive index was formed of five items that asked the extent to which participants felt “nervous,” “tense,” “worried,” “threatened,” or “anxious”; based in part on (*51*)], whether people felt listened to by their partner (an additive index was formed of four items that asked the extent to which participants agreed they felt “listened to,” “heard,” “understood,” and “seen”), and warmth felt toward their partner (“How do you feel toward your conversational partner? Please rate your feelings on a scale of 0 to 100, where 0 means the most unfavorable/cold, and 100 means the most favorable/warmest.”). We asked the manipulation check items, the mechanism items, one behavioral intention item, and the respect items of the participants who indicated that they had a conversation.

### Analytical approach

We used the same analytical approach as detailed in study 1: We used linear regressions with indicators for treatments, with the placebo condition as a referent. (Figure S6 reports the comparison between the Inparty Strengths versus Outparty Flaws conditions, as registered. Last, in post hoc tests discussed below and reported in fig. S5, we collapsed across the two conditions discussing partisanship preferences and compared them to the Perfect Day and Placebo conditions. Of note, we adjusted these *P* values separately, but including the relevant *P* values when calculating this adjustment.) We always use clustered standard errors (*29*). The regressions include the following covariates: the baseline value of the variable where available, age, education, gender, race/ethnicity, party identification, and ideology (the last three were added post hoc to be consistent with study 1). We follow the same approach for adjusting our *P* values for multiple comparisons (*30*), and we again rescaled all dependent variables to SD one, so all estimates we report are in SDs.

#### Exclusions

We used the same exclusions as in study 1 with the following exceptions: We use whether participants clicked the survey page where the link appeared, rather than appearing on the landing page, to help determine whether a participant was treated; we excluded a small number of participants who were paired with partners in a different treatment condition due to a coding error; and we excluded participants who had participated in a logistical pilot we conducted and we allowed to participate in the study erroneously.

We determined all of these exclusion criteria seeking to be as consistent as possible with study 1. Figures S1 and S3 provide a graphical overview of the recruitment process and exclusion criteria.

## Supplementary Materials

### This PDF file includes:

Figs. S1 to S7 Tables S1 to S9 References
