# Supplementary material for: The promise and pitfalls of cross-partisan conversations for reducing affective polarization: Evidence from randomized experiments
Source: Sci Adv. 2022 Jun 22;8(25):eabn5515. doi: 10.1126/sciadv.abn5515 (PMC9217089; doi:10.1126/sciadv.abn5515)
Supplement: Supplementary file 1 — Figs. S1 to S7 Tables S1 to S9 References [file sciadv.abn5515_sm.pdf]

Science Advances
AAAS

Supplementary Materials for

The promise and pitfalls of cross-partisan conversations for reducing affective polarization: Evidence from randomized experiments

Erik Santoro* and David E. Broockman*

Corresponding author: Erik Santoro, esantoro@stanford.edu; David E. Broockman, dbroockman@berkeley.edu

Sci. Adv. **8**, eabn5515 (2022)
DOI: 10.1126/sciadv.abn5515

This PDF file includes:

Figs. S1 to S7
Tables S1 to S9
References

A SM Figures and Tables

Figure S1: Summary of Study 1 and Study 2, Part 2 Methods and Exclusions

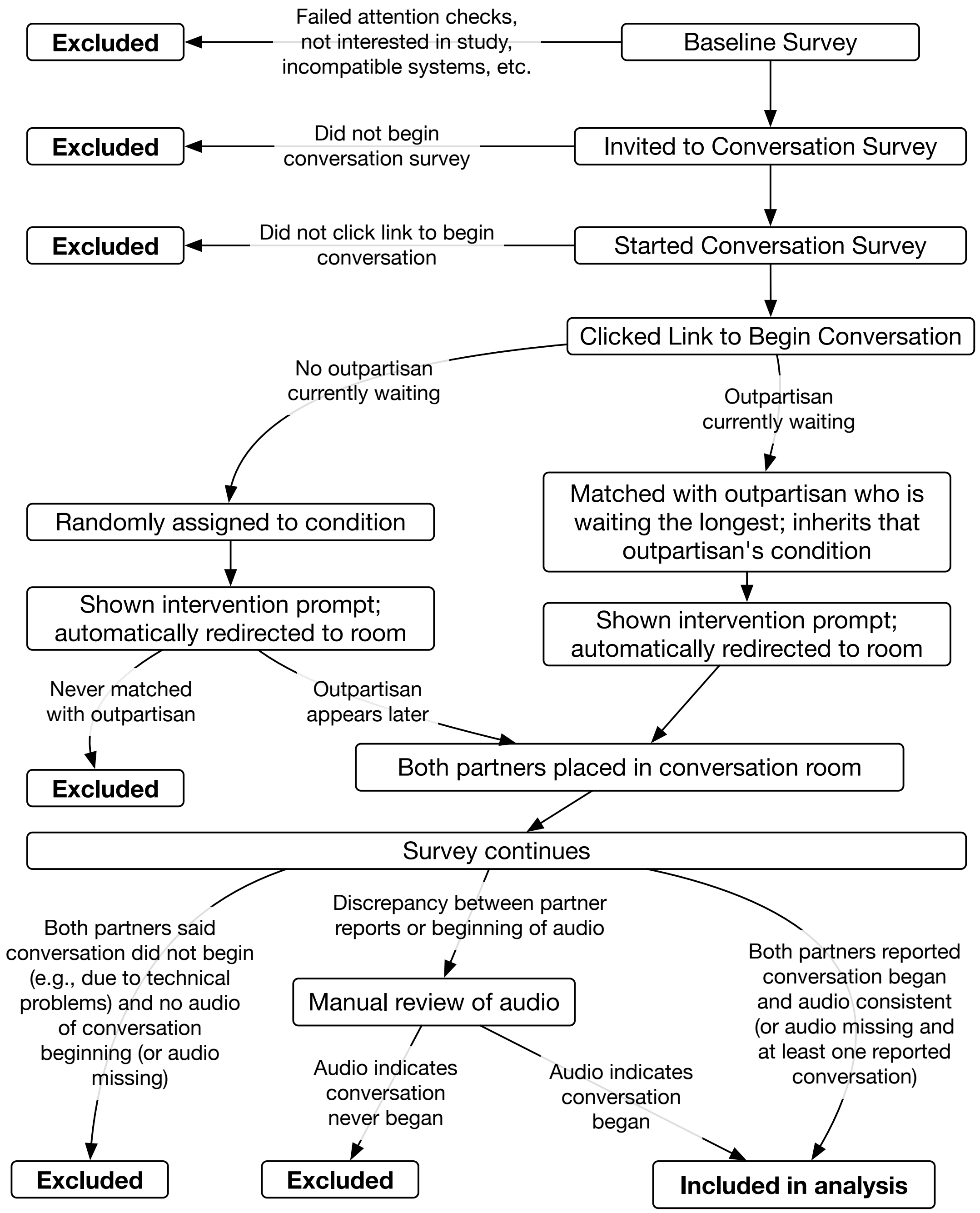

Figure S2: Study 1: Additional Results

(a) Potential Mechanisms

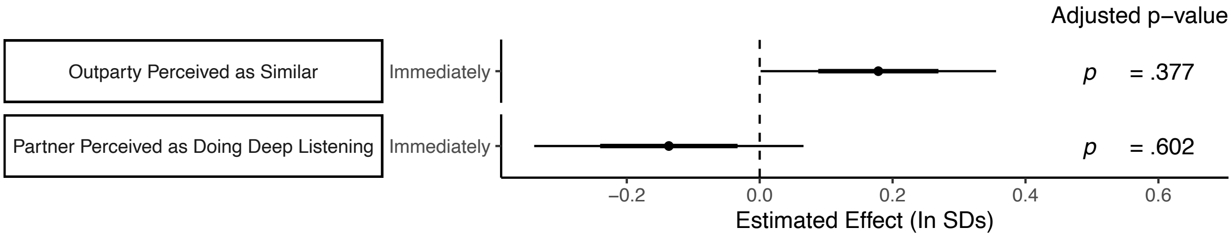

Notes: Points show the estimated effects of the Perfect Day condition in Study 1. Standard errors (thick lines) and 95% confidence intervals (thin lines) surround the point estimates. Adjusted p-values are adjusted across all measures besides the primary outcomes, which are not adjusted as described in the text.

(b) Moderators

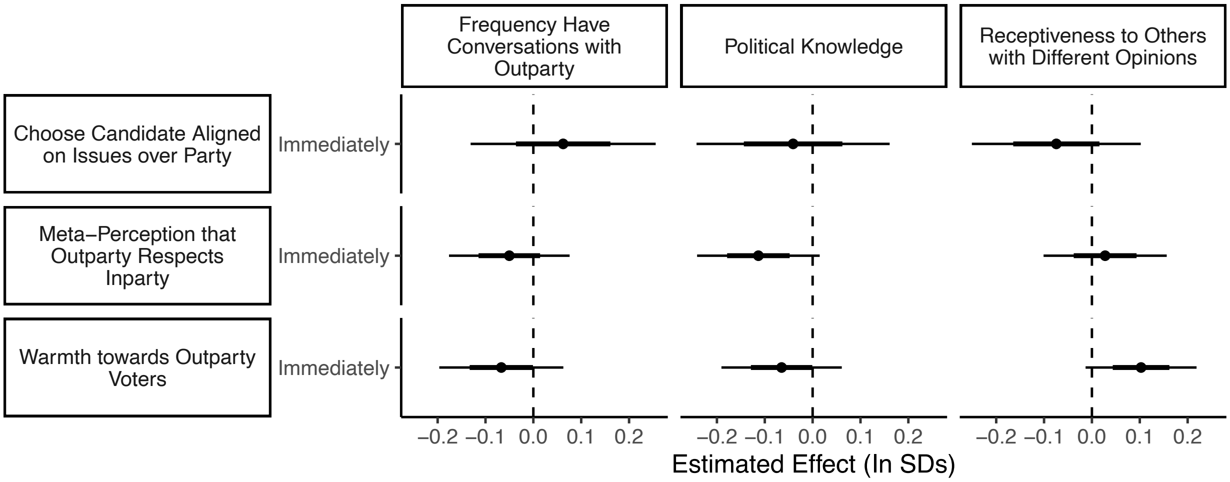

Notes: Points show the coefficient on an interaction term between the Perfect Day condition and the moderator shown in the labels at top. The labels at right show the dependent variable. Standard errors (thick lines) and 95% confidence intervals (thin lines) surround the point estimates. Adjusted p-values are adjusted among all non-primary hypothesis tests.

Figure S3: Summary of Study 2, Part 1 Methods and Exclusions

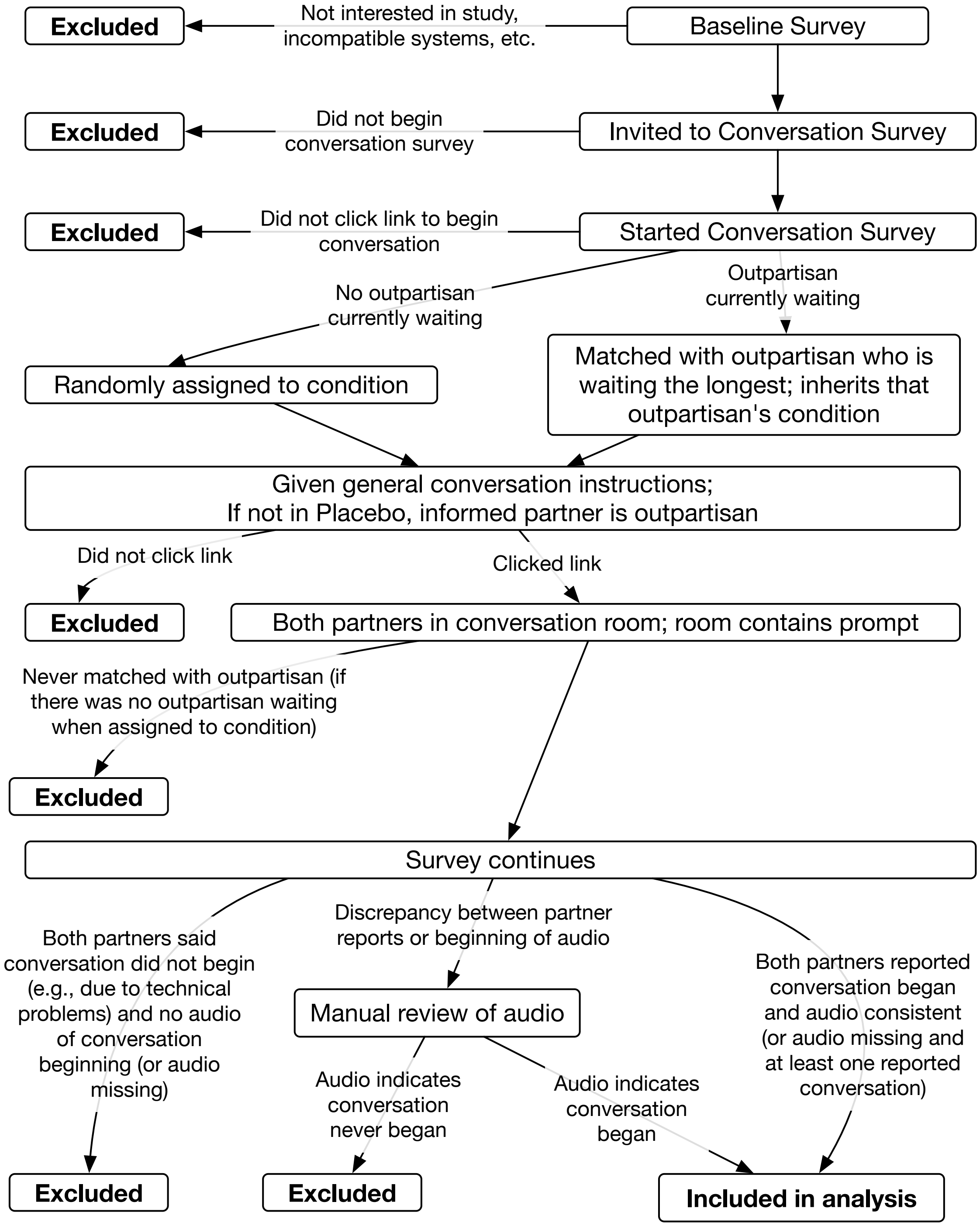

Figure S4: Study 2: Additional Results

(a) Potential Mechanisms

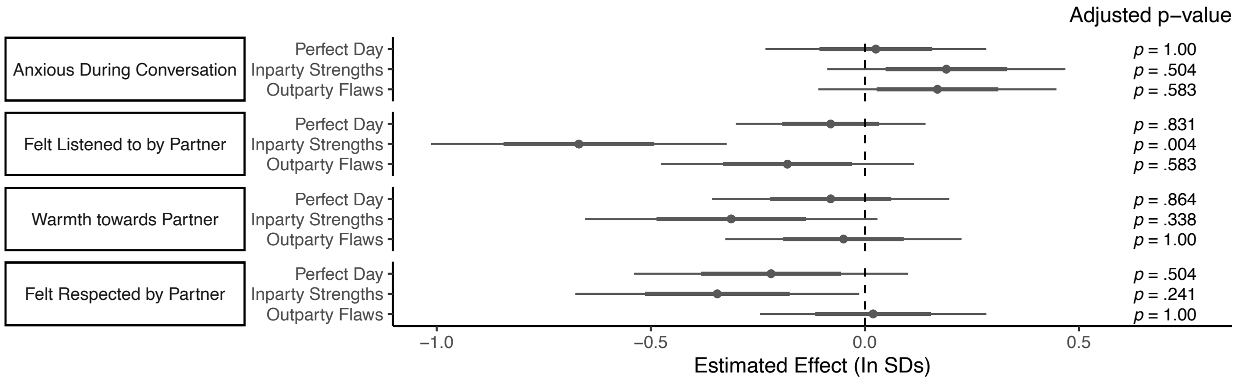

Notes: Points show the estimated effects of each condition in Study 2. Standard errors (thick lines) and 95% confidence intervals (thin lines) surround the point estimates. Adjusted p-values are adjusted across all measures, including mechanisms, besides primary outcomes.

(b) Moderators

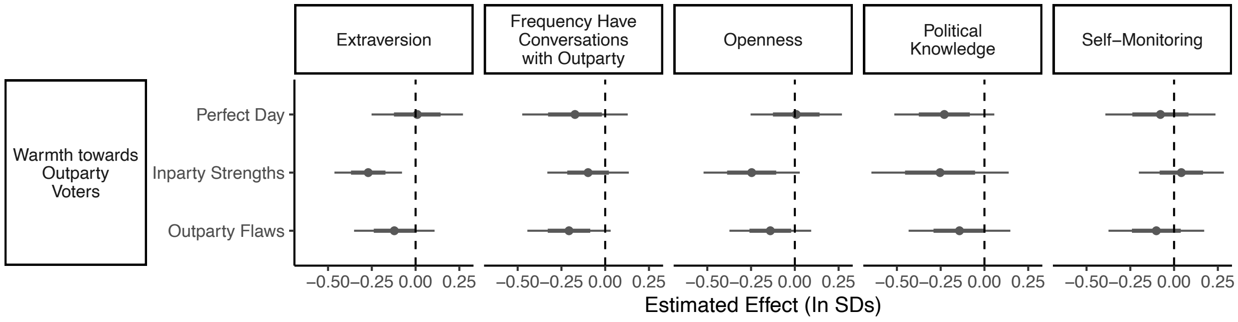

Notes: Points show the coefficient on an interaction term between each condition (left) and the moderator (top). Standard errors (thick lines) and 95% confidence intervals (thin lines) surround the point estimates. Adjusted p-values are adjusted among all measures, including moderators, besides primary outcomes.

Figure S5: Study 2: Pooled effects of either Inparty Strength or Outparty Flaws condition

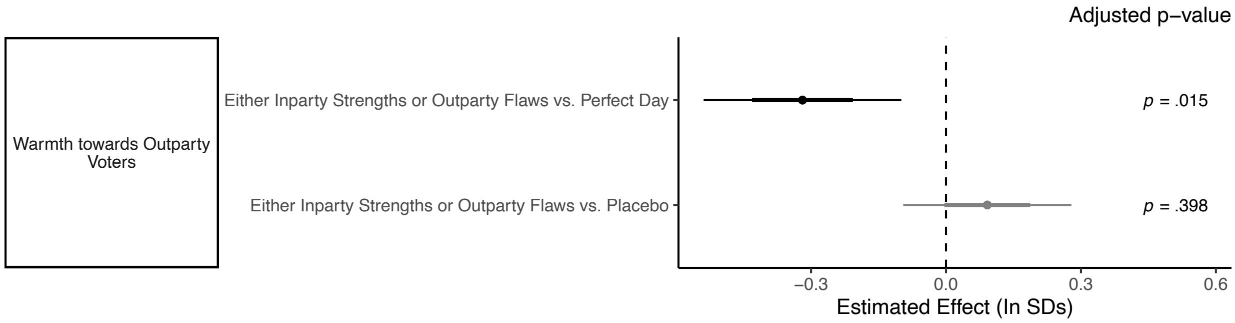

Points show the estimated effects of either the Inparty Strengths or Outparty Flaws condition (pooled) in Study 2. Standard errors (thick lines) and 95% confidence intervals (thin lines) surround the point estimates. Adjusted p*-values are adjusted among all post-hoc comparisons. The first coefficient shows the effects of either condition (pooled) versus the Perfect Day group. The second coefficient shows the effects of either condition (pooled) versus the Placebo group.*

Figure S6: Study 2: Comparing Effects of Inparty Strengths vs. Outparty Flaws Conditions

(a) Manipulation Checks

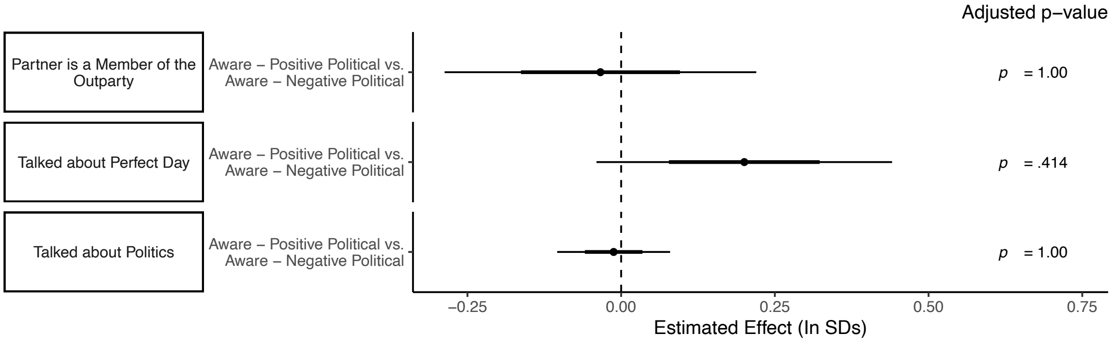

(b) Intergroup Attitudes

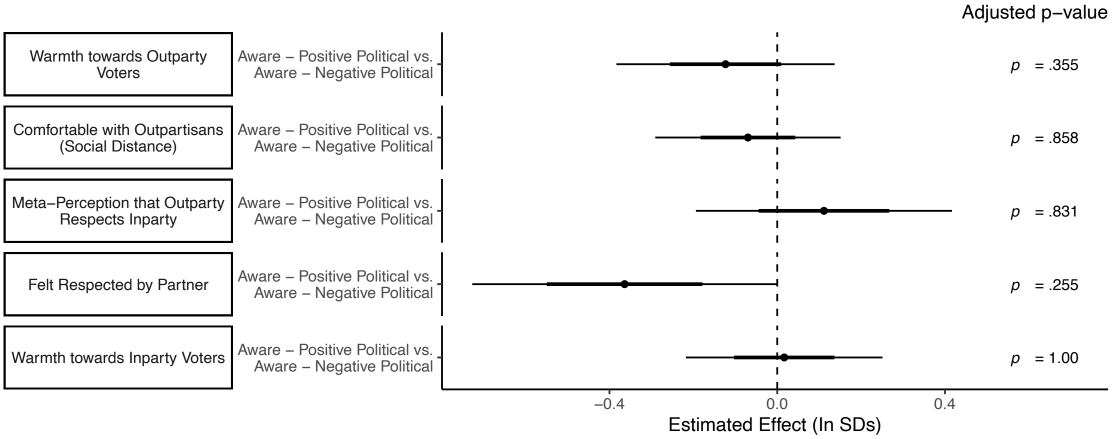

(c) Outcomes Relevant to Democratic Accountability

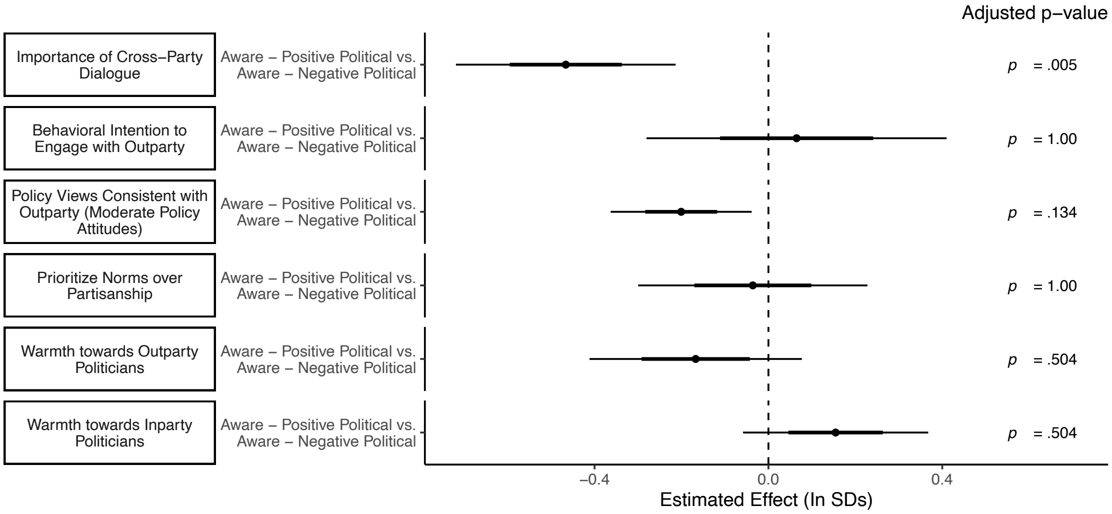

Notes: Points show the estimated differences between the effects of the Inparty Strengths and Outparty Flaws conditions in Study 2. Standard errors (thick lines) and 95% confidence intervals (thin lines) surround the point estimates. Adjusted p-values are adjusted across all non-primary outcomes using the procedure outlined in (30). As described in the text, p-values for primary outcomes are not adjusted.

Figure S7: Raw Means of Warthm Towards Outparty, with 95% Confidence Intervals

(a) Study 1

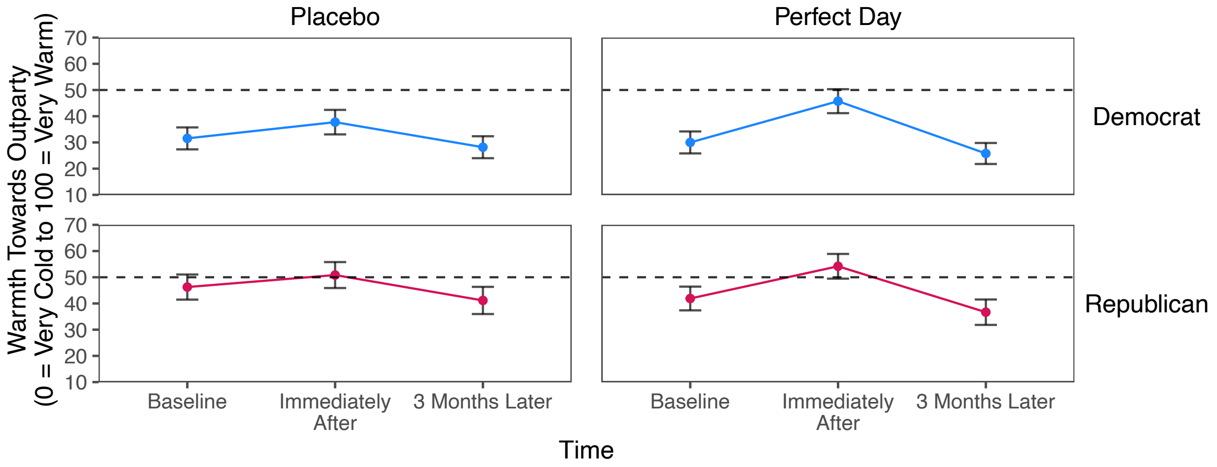

(b) Study 2

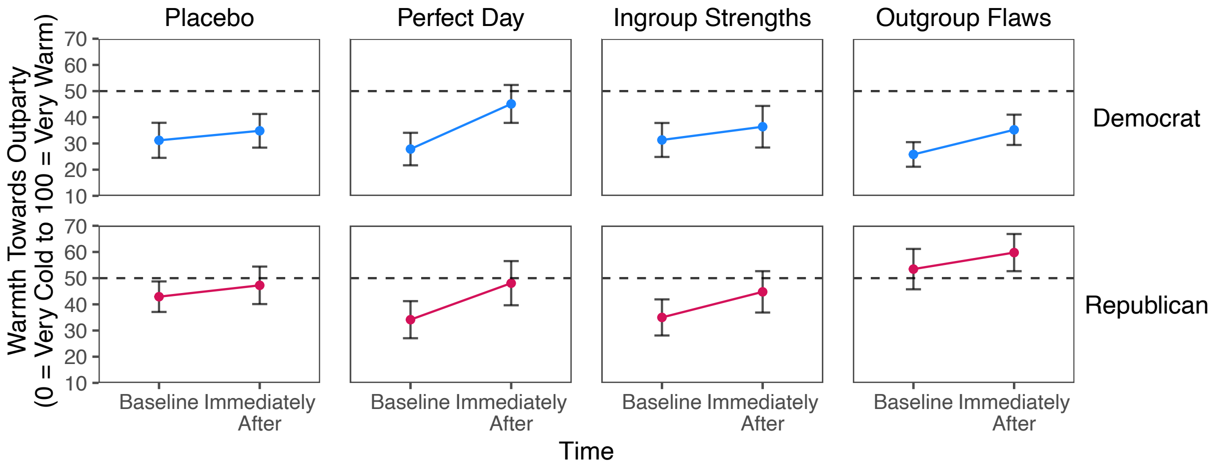

Notes: Note that because the data are repeated observations of the same individuals, the 95% confidence intervals shown in the Figure cannot be used to assess the strength of the statistical evidence for differences between time points or between conditions.

Table S1: Study 1: Demographic Composition of Samples

	Completed Screener	Entered Conversation Room	Began Conversation (Used in Analyses)†			Started Follow-Up Survey (Used in Follow-Up Survey Analyses)‡		
			All	Placebo	Perfect Day	All	Placebo	Perfect Day
N	7,904	986	478	218	260	419	184	235
Education: College or post-graduate	-	61.26%	62.13%	59.17%	64.62%	62.77%	60.87%	64.26%
Education: High school or less	-	9.43%	8.79%	11.47%	6.54%	8.11%	9.78%	6.81%
Education: Some college	-	29.31%	29.08%	29.36%	28.85%	29.12%	29.35%	28.94%
Race/Ethnicity: African American	-	13.79%	10.04%	9.17%	10.77%	7.88%	7.61%	8.09%
Race/Ethnicity: Asian	-	7.81%	7.11%	9.63%	5%	6.92%	9.78%	4.68%
Race/Ethnicity: Hispanic/Latino	-	4.06%	4.18%	3.21%	5%	4.3%	2.72%	5.53%
Race/Ethnicity: Multiple Selected	-	5.38%	5.44%	5.5%	5.38%	5.49%	4.89%	5.96%
Race/Ethnicity: Native American	-	0.3%	0 %	0 %	0 %	0 %	0 %	0 %
Race/Ethnicity: Other	-	0.71%	0.42%	0 %	0.77%	0.48%	0 %	0.85%
Race/Ethnicity: Pacific Islander	-	0.2%	0.21%	0 %	0.38%	0.24%	0 %	0.43%
Race/Ethnicity: White	-	67.75%	72.59%	72.48%	72.69%	74.7%	75%	74.47%
Gender: Female	-	49.29%	50.63%	57.8%	44.62%	51.79%	61.41%	44.26%
Gender: Male	-	49.59%	48.12%	41.28%	53.85%	47.26%	38.04%	54.47%
Gender: Prefer to self describe	-	1.12%	1.26%	0.92%	1.54%	0.95%	0.54%	1.28%
Age (mean), in years	-	38.68	39.99	39.59	40.33	40.95	40.88	41.00
Party identification: Closer to Democratic Party	13.27%	13.08%	11.3%	14.22%	8.85%	10.98%	13.59%	8.94%
Party identification: Closer to Republican Party	6.24%	9.63%	12.34%	11.93%	12.69%	11.46%	9.78%	12.77%
Party identification: Not very strong Democrat	18.27%	18.46%	14.85%	15.6%	14.23%	15.27%	15.22%	15.32%
Party identification: Not very strong Republican	9.03%	13.59%	18.41%	20.18%	16.92%	18.85%	20.65%	17.45%
Party identification: Strong Democrat	30.69%	26.88%	23.85%	20.64%	26.54%	23.63%	21.2%	25.53%
Party identification: Strong Republican	11.75%	18.26%	19.25%	17.43%	20.77%	19.81%	19.57%	20%
Party identification: Neither	10.74%	0 %	0 %	0 %	0 %	0 %	0 %	0 %
Party identification: Not Listed	0.03%	0.1%	0 %	0 %	0 %	0 %	0 %	0 %

Notes: This Table shows the composition of the sample at every step across selected covariates. Some covariates (e.g., education) are not available for the screener because these demographics were only asked about at the beginning of the conversation survey and were not asked on the screener.

†: A joint test for differences between conditions on demographics in the table was just past the threshold for significance ($p = 0.05$) due to the difference in gender across the conditions discussed in the text. As discussed in the text, men were slightly over-represented in the *Perfect Day* condition, perhaps indicating that men were especially interested in having the *Perfect Day* conversation; however, gender is not a significant predictor of our outcomes and we find similar effect estimates for men and women on the items where we did find effects. Removing gender, the test is no longer significant ($p = 0.24$).

‡: Similar to the data from the main conversation survey, we found slight differences between conditions on gender, although a joint test for differences between conditions fell just short of significance ($p = 0.05$). Removing gender, the test is no longer significant.

Table S2: Study 2: Demographic Composition of Samples

	Completed Screener and Provided Email	Clicked Page Containing Link to Conversation	Began Conversation (Used in Analyses)†				
			All	Placebo	Perfect Day	Inparty Strengths	Outparty Flaws
N	2,541	607	338	100	74	83	81
Education: College or post-graduate	63.72%	70.68%	72.19%	74%	72.97%	69.88%	71.6%
Education: High school or less	8.42%	7.25%	6.21%	6%	5.41%	6.02%	7.41%
Education: Some college	27.86%	22.08%	21.6%	20%	21.62%	24.1%	20.99%
Race/Ethnicity: African American	3.74%	2.64%	2.66%	6%	2.7%	0 %	1.23%
Race/Ethnicity: Asian	5.94%	7.91%	7.99%	7%	8.11%	8.43%	8.64%
Race/Ethnicity: Hispanic/Latino	3.19%	3.13%	2.96%	3%	2.7%	2.41%	3.7%
Race/Ethnicity: Multiple Selected	6.77%	7.74%	7.1%	5%	5.41%	13.25%	4.94%
Race/Ethnicity: Native American	0.35%	0.16%	0.3%	0 %	0 %	1.2%	0 %
Race/Ethnicity: Not Listed	0.04%	0 %	0 %	0 %	0 %	0 %	0 %
Race/Ethnicity: Other	1.65%	1.98%	1.78%	1%	2.7%	3.61%	0 %
Race/Ethnicity: Pacific Islander	0.16%	0 %	0 %	0 %	0 %	0 %	0 %
Race/Ethnicity: White	78.16%	76.44%	77.22%	78%	78.38%	71.08%	81.48%
Gender: Female	56.67%	51.73%	48.52%	44%	45.95%	50.6%	54.32%
Gender: Male	42.07%	47.45%	51.18%	56%	54.05%	48.19%	45.68%
Gender: Not Listed	0.12%	0 %	0 %	0 %	0 %	0 %	0 %
Gender: Prefer to self describe	1.14%	0.82%	0.3%	0 %	0 %	1.2%	0 %
Age (mean), years	45.47	44.55	43.63	44.15	41.93	42.81	45.38
Party identification: Closer to Democratic Party	17.24%	14.66%	13.31%	12%	16.22%	19.28%	6.17%
Party identification: Closer to Republican Party	9.09%	12.69%	15.38%	12%	18.92%	14.46%	17.28%
Party identification: Not very strong Democrat	13.7%	10.21%	9.17%	10%	5.41%	8.43%	12.35%
Party identification: Not very strong Republican	8.5%	12.69%	15.38%	14%	14.86%	15.66%	17.28%
Party identification: Strong Democrat	38.17%	32.78%	28.99%	28%	29.73%	25.3%	33.33%
Party identification: Strong Republican	13.3%	16.97%	17.75%	24%	14.86%	16.87%	13.58%

Notes: This Table shows the composition of the sample at every step across selected covariates.

†: A joint test for differences between conditions on demographics in the table is insignificant ($p = 0.44$).

Table S3: Numerical Results and Variable Classification: Study 1

Category	Measure Name	Time Period	DV Type	Estimate
Democratic Accountability	Choose Candidate Aligned on Issues over Party	Immediately	Primary	-0.073
Democratic Accountability	Likelihood of Talking to Outparty Neighbor about Politics	Immediately	Secondary	-0.048
Democratic Accountability	Policy Views Consistent with Outparty (Moderate Policy Attitudes)	Immediately	Secondary	-0.019
Democratic Accountability	Prioritize Norms over Partisanship	Immediately	Secondary	-0.009
Democratic Accountability	Request to Subscribe to Bipartisan Newsletter	Immediately	Secondary	-0.04
Democratic Accountability	Support for Bipartisanship	Immediately	Secondary	-0.235
Democratic Accountability	Choose Candidate Aligned on Issues over Party	3 Months After	Post-Hoc	-0.093
Democratic Accountability	Importance of Cross-Party Dialogue	3 Months After	Post-Hoc	-0.068
Democratic Accountability	Policy Views Consistent with Outparty (Moderate Policy Attitudes)	3 Months After	Post-Hoc	0.011
Democratic Accountability	Warmth towards Inparty Politicians	3 Months After	Post-Hoc	0.116
Democratic Accountability	Warmth towards Outparty Politicians	3 Months After	Post-Hoc	0.03
Democratic Accountability	Warmth towards Inparty Politicians	Immediately	Tertiary	0.036

Table S3: Numerical Results and Variable Classification: Study 1 (Continued)

Category	Measure Name	Time Period	DV Type	Estimate
Democratic Accountability	Warmth towards Outparty Politicians	Immediately	Tertiary	0.12
Intergroup	Meta-Perception that Outparty Respects Inparty	Immediately	Primary	0.457
Intergroup	Warmth towards Outparty Voters	Immediately	Primary	0.34
Intergroup	Humanization of Outparty	Immediately	Secondary	-0.021
Intergroup	Meta-Perception that Outparty Respects Inparty	3 Months After	Post-Hoc	0.136
Intergroup	Warmth towards Inparty Voters	3 Months After	Post-Hoc	0.029
Intergroup	Warmth towards Outparty Voters	3 Months After	Post-Hoc	-0.021
Intergroup	Warmth towards Inparty Voters	Immediately	Tertiary	0.078
Manipulation Check	Partner is a Member of the Outparty	Immediately	Manipulation Check	0.81
Manipulation Check	Talked about Perfect Day	Immediately	Manipulation Check	-0.104
Manipulation Check	Talked about Politics	Immediately	Manipulation Check	0.361
Manipulation Check	Talked about Unrelated Topics	Immediately	Manipulation Check	-0.001
Mechanism	Outparty Perceived as Similar	Immediately	Mechanism	0.179
Mechanism	Partner Perceived as Doing Deep Listening	Immediately	Mechanism	-0.137
Moderator	Moderation of Choose Candidate Aligned on Issues over Party by Frequency Have Conversations with Outparty	Immediately	Moderator	0.062

Table S3: Numerical Results and Variable Classification: Study 1 (Continued)

Category	Measure Name	Time Period	DV Type	Estimate
Moderator	Moderation of Choose Candidate Aligned on Issues over Party by Political Knowledge	Immediately	Moderator	-0.041
Moderator	Moderation of Choose Candidate Aligned on Issues over Party by Receptiveness to Others with Different Opinions	Immediately	Moderator	-0.074
Moderator	Moderation of Meta-Perception that Outparty Respects Inparty by Frequency Have Conversations with Outparty	Immediately	Moderator	-0.05
Moderator	Moderation of Meta-Perception that Outparty Respects Inparty by Political Knowledge	Immediately	Moderator	-0.113
Moderator	Moderation of Meta-Perception that Outparty Respects Inparty by Receptiveness to Others with Different Opinions	Immediately	Moderator	0.028
Moderator	Moderation of Warmth towards Outparty Voters by Frequency Have Conversations with Outparty	Immediately	Moderator	-0.067
Moderator	Moderation of Warmth towards Outparty Voters by Political Knowledge	Immediately	Moderator	-0.065

Table S3: Numerical Results and Variable Classification: Study 1 (Continued)

Category	Measure Name	Time Period	DV Type	Estimate
Moderator	Moderation of Warmth towards Outparty Voters by Receptiveness to Others with Different Opinions	Immediately	Moderator	0.103

Notes: All results are the estimated effect of the Perfect Day condition versus the Placebo. p-values below 0.001 are rounded to 0. As described in the text, p-values for primary outcomes are

Table S4: Numerical Results and Variable Classification: Study 2

Category	Measure Name	DV Type	Condition Comparison	Estimate	SE	t	Unadjusted p-value	Adjusted p-value
Democratic Accountability	Behavioral Intention to Engage with Outparty	Secondary	Outparty Flaws vs. Placebo	-0.011	0.149	-0.071	0.943	1
Democratic Accountability	Behavioral Intention to Engage with Outparty	Secondary	Perfect Day vs. Placebo	0.111	0.154	0.723	0.472	0.831
Democratic Accountability	Behavioral Intention to Engage with Outparty	Secondary	Inparty Strengths vs. Outparty Flaws	0.065	0.176	0.368	0.714	1
Democratic Accountability	Behavioral Intention to Engage with Outparty	Secondary	Inparty Strengths vs. Placebo	0.054	0.163	0.333	0.74	1
Democratic Accountability	Importance of Cross-Party Dialogue	Secondary	Outparty Flaws vs. Placebo	0.369	0.101	3.662	0	0.005
Democratic Accountability	Importance of Cross-Party Dialogue	Secondary	Perfect Day vs. Placebo	-0.096	0.124	-0.771	0.443	0.831
Democratic Accountability	Importance of Cross-Party Dialogue	Secondary	Inparty Strengths vs. Outparty Flaws	-0.466	0.129	-3.615	0.001	0.005
Democratic Accountability	Importance of Cross-Party Dialogue	Secondary	Inparty Strengths vs. Placebo	-0.097	0.11	-0.884	0.379	0.831
Democratic Accountability	Policy Views Consistent with Outparty (Moderate Policy Attitudes)	Secondary	Outparty Flaws vs. Placebo	0.166	0.073	2.26	0.026	0.172
Democratic Accountability	Policy Views Consistent with Outparty (Moderate Policy Attitudes)	Secondary	Perfect Day vs. Placebo	0.046	0.075	0.608	0.545	0.858

Table S4: Numerical Results and Variable Classification: Study 2 (Continued)

Category	Measure Name	DV Type	Condition Comparison	Estimate	SE	t	Unadjusted p-value	Adjusted p-value
Democratic Accountability	Policy Views Consistent with Outparty (Moderate Policy Attitudes)	Secondary	Inparty Strengths vs. Outparty Flaws	-0.201	0.083	-2.424	0.018	0.134
Democratic Accountability	Policy Views Consistent with Outparty (Moderate Policy Attitudes)	Secondary	Inparty Strengths vs. Placebo	-0.035	0.073	-0.476	0.635	0.982
Democratic Accountability	Prioritize Norms over Partisanship	Secondary	Outparty Flaws vs. Placebo	-0.229	0.124	-1.848	0.068	0.317
Democratic Accountability	Prioritize Norms over Partisanship	Secondary	Perfect Day vs. Placebo	-0.144	0.104	-1.377	0.172	0.504
Democratic Accountability	Prioritize Norms over Partisanship	Secondary	Inparty Strengths vs. Outparty Flaws	-0.036	0.135	-0.267	0.79	1
Democratic Accountability	Prioritize Norms over Partisanship	Secondary	Inparty Strengths vs. Placebo	-0.265	0.121	-2.196	0.031	0.189
Democratic Accountability	Warmth towards Inparty Politicians	Tertiary	Outparty Flaws vs. Placebo	0.007	0.098	0.07	0.944	1
Democratic Accountability	Warmth towards Inparty Politicians	Tertiary	Perfect Day vs. Placebo	-0.023	0.093	-0.245	0.807	1
Democratic Accountability	Warmth towards Inparty Politicians	Tertiary	Inparty Strengths vs. Outparty Flaws	0.155	0.109	1.423	0.159	0.504
Democratic Accountability	Warmth towards Inparty Politicians	Tertiary	Inparty Strengths vs. Placebo	0.162	0.092	1.75	0.084	0.338
Democratic Accountability	Warmth towards Outparty Politicians	Tertiary	Outparty Flaws vs. Placebo	0.024	0.11	0.223	0.824	1

Table S4: Numerical Results and Variable Classification: Study 2 (Continued)

Category	Measure Name	DV Type	Condition Comparison	Estimate	SE	t	Unadjusted p-value	Adjusted p-value
Democratic Accountability	Warmth towards Outparty Politicians	Tertiary	Perfect Day vs. Placebo	0.151	0.12	1.258	0.212	0.562
Democratic Accountability	Warmth towards Outparty Politicians	Tertiary	Inparty Strengths vs. Outparty Flaws	-0.167	0.125	-1.343	0.183	0.504
Democratic Accountability	Warmth towards Outparty Politicians	Tertiary	Inparty Strengths vs. Placebo	-0.143	0.106	-1.348	0.181	0.504
Intergroup	Warmth towards Outparty Voters	Primary	Outparty Flaws vs. Placebo	0.148	0.115	1.29	0.201	0.201
Intergroup	Warmth towards Outparty Voters	Primary	Perfect Day vs. Placebo	0.406	0.116	3.511	0.001	0.001
Intergroup	Warmth towards Outparty Voters	Primary	Inparty Strengths vs. Outparty Flaws	-0.123	0.133	-0.929	0.355	0.355
Intergroup	Warmth towards Outparty Voters	Primary	Inparty Strengths vs. Placebo	0.025	0.117	0.214	0.831	0.831
Intergroup	Comfortable with Outpartisans (Social Distance)	Secondary	Outparty Flaws vs. Placebo	0.034	0.091	0.377	0.707	1
Intergroup	Comfortable with Outpartisans (Social Distance)	Secondary	Perfect Day vs. Placebo	-0.079	0.086	-0.912	0.364	0.831
Intergroup	Comfortable with Outpartisans (Social Distance)	Secondary	Inparty Strengths vs. Outparty Flaws	-0.07	0.113	-0.62	0.537	0.858

Table S4: Numerical Results and Variable Classification: Study 2 (Continued)

Category	Measure Name	DV Type	Condition Comparison	Estimate	SE	t	Unadjusted p-value	Adjusted p-value
Intergroup	Comfortable with Outpartisans (Social Distance)	Secondary	Inparty Strengths vs. Placebo	-0.036	0.107	-0.333	0.74	1
Intergroup	Meta-Perception that Outparty Respects Inparty	Secondary	Outparty Flaws vs. Placebo	0.182	0.128	1.422	0.159	0.504
Intergroup	Meta-Perception that Outparty Respects Inparty	Secondary	Perfect Day vs. Placebo	0.078	0.136	0.573	0.568	0.864
Intergroup	Meta-Perception that Outparty Respects Inparty	Secondary	Inparty Strengths vs. Outparty Flaws	0.111	0.156	0.715	0.477	0.831
Intergroup	Meta-Perception that Outparty Respects Inparty	Secondary	Inparty Strengths vs. Placebo	0.293	0.136	2.157	0.034	0.195
Intergroup	Warmth towards Inparty Voters	Tertiary	Outparty Flaws vs. Placebo	0.124	0.123	1.008	0.316	0.709
Intergroup	Warmth towards Inparty Voters	Tertiary	Perfect Day vs. Placebo	0.13	0.107	1.216	0.228	0.583
Intergroup	Warmth towards Inparty Voters	Tertiary	Inparty Strengths vs. Outparty Flaws	0.017	0.12	0.14	0.889	1
Intergroup	Warmth towards Inparty Voters	Tertiary	Inparty Strengths vs. Placebo	0.141	0.103	1.367	0.175	0.504
Intergroup	Warmth towards Outparty Voters	Primary (Unregistered)	Either Inparty Strengths or Outparty Flaws vs. Placebo	0.092	0.095	0.962	0.338	0.398
Intergroup	Warmth towards Outparty Voters	Primary (Unregistered)	Either Inparty Strengths or Outparty Flaws vs. Perfect Day	-0.319	0.112	-2.846	0.006	0.015

Table S4: Numerical Results and Variable Classification: Study 2 (Continued)

Category	Measure Name	DV Type	Condition Comparison	Estimate	SE	t	Unadjusted p-value	Adjusted p-value
Manipulation Check	Partner is a Member of the Outparty	Manipulation Check	Outparty Flaws vs. Placebo	1.271	0.11	11.552	0	0.001
Manipulation Check	Partner is a Member of the Outparty	Manipulation Check	Perfect Day vs. Placebo	0.257	0.128	2.003	0.049	0.246
Manipulation Check	Partner is a Member of the Outparty	Manipulation Check	Inparty Strengths vs. Outparty Flaws	-0.034	0.129	-0.261	0.795	1
Manipulation Check	Partner is a Member of the Outparty	Manipulation Check	Inparty Strengths vs. Placebo	1.238	0.119	10.42	0	0.001
Manipulation Check	Talked about Perfect Day	Manipulation Check	Outparty Flaws vs. Placebo	-1.773	0.087	-20.303	0	0.001
Manipulation Check	Talked about Perfect Day	Manipulation Check	Perfect Day vs. Placebo	0.022	0.058	0.379	0.706	1
Manipulation Check	Talked about Perfect Day	Manipulation Check	Inparty Strengths vs. Outparty Flaws	0.2	0.123	1.633	0.106	0.414
Manipulation Check	Talked about Perfect Day	Manipulation Check	Inparty Strengths vs. Placebo	-1.572	0.105	-14.983	0	0.001
Manipulation Check	Talked about Politics	Manipulation Check	Outparty Flaws vs. Placebo	1.767	0.086	20.538	0	0.001
Manipulation Check	Talked about Politics	Manipulation Check	Perfect Day vs. Placebo	0.088	0.124	0.716	0.476	0.831
Manipulation Check	Talked about Politics	Manipulation Check	Inparty Strengths vs. Outparty Flaws	-0.012	0.047	-0.26	0.795	1
Manipulation Check	Talked about Politics	Manipulation Check	Inparty Strengths vs. Placebo	1.755	0.087	20.086	0	0.001

Table S4: Numerical Results and Variable Classification: Study 2 (Continued)

Category	Measure Name	DV Type	Condition Comparison	Estimate	SE	t	Unadjusted p-value	Adjusted p-value
Mechanism	Warmth towards Partner	Secondary	Outparty Flaws vs. Placebo	-0.05	0.141	-0.354	0.724	1
Mechanism	Warmth towards Partner	Secondary	Perfect Day vs. Placebo	-0.08	0.141	-0.564	0.574	0.864
Mechanism	Warmth towards Partner	Secondary	Inparty Strengths vs. Outparty Flaws	-0.262	0.196	-1.341	0.184	0.504
Mechanism	Warmth towards Partner	Secondary	Inparty Strengths vs. Placebo	-0.312	0.174	-1.79	0.077	0.338
Mechanism	Anxious During Conversation	Mechanism	Outparty Flaws vs. Placebo	0.17	0.142	1.195	0.235	0.583
Mechanism	Anxious During Conversation	Mechanism	Perfect Day vs. Placebo	0.026	0.132	0.195	0.846	1
Mechanism	Anxious During Conversation	Mechanism	Inparty Strengths vs. Outparty Flaws	0.021	0.168	0.124	0.902	1
Mechanism	Anxious During Conversation	Mechanism	Inparty Strengths vs. Placebo	0.19	0.142	1.342	0.183	0.504
Mechanism	Felt Listened to by Partner	Mechanism	Outparty Flaws vs. Placebo	-0.181	0.151	-1.199	0.234	0.583
Mechanism	Felt Listened to by Partner	Mechanism	Perfect Day vs. Placebo	-0.08	0.113	-0.705	0.483	0.831
Mechanism	Felt Listened to by Partner	Mechanism	Inparty Strengths vs. Outparty Flaws	-0.487	0.207	-2.354	0.021	0.149
Mechanism	Felt Listened to by Partner	Mechanism	Inparty Strengths vs. Placebo	-0.668	0.176	-3.793	0	0.004

Table S4: Numerical Results and Variable Classification: Study 2 (Continued)

Category	Measure Name	DV Type	Condition Comparison	Estimate	SE	t	Unadjusted p-value	Adjusted p-value
Mechanism	Felt Respected by Partner	Mechanism	Outparty Flaws vs. Placebo	0.019	0.135	0.143	0.886	1
Mechanism	Felt Respected by Partner	Mechanism	Perfect Day vs. Placebo	-0.219	0.163	-1.341	0.184	0.504
Mechanism	Felt Respected by Partner	Mechanism	Inparty Strengths vs. Outparty Flaws	-0.364	0.185	-1.964	0.053	0.255
Mechanism	Felt Respected by Partner	Mechanism	Inparty Strengths vs. Placebo	-0.345	0.169	-2.038	0.045	0.241
Moderator	Moderation of Warmth towards Outparty Voters by Extraversion	Moderator	Outparty Flaws vs. Placebo	-0.121	0.117	-1.035	0.304	0.695
Moderator	Moderation of Warmth towards Outparty Voters by Extraversion	Moderator	Perfect Day vs. Placebo	0.01	0.133	0.072	0.943	1
Moderator	Moderation of Warmth towards Outparty Voters by Extraversion	Moderator	Inparty Strengths vs. Outparty Flaws	-0.149	0.131	-1.137	0.261	0.594
Moderator	Moderation of Warmth towards Outparty Voters by Extraversion	Moderator	Inparty Strengths vs. Placebo	-0.271	0.098	-2.748	0.008	0.065

Table S4: Numerical Results and Variable Classification: Study 2 (Continued)

Category	Measure Name	DV Type	Condition Comparison	Estimate	SE	t	Unadjusted p-value	Adjusted p-value
Moderator	Moderation of Warmth towards Outparty Voters by Frequency Have Conversations with Outparty	Moderator	Outparty Flaws vs. Placebo	-0.207	0.121	-1.704	0.094	0.372
Moderator	Moderation of Warmth towards Outparty Voters by Frequency Have Conversations with Outparty	Moderator	Perfect Day vs. Placebo	-0.173	0.154	-1.123	0.267	0.594
Moderator	Moderation of Warmth towards Outparty Voters by Frequency Have Conversations with Outparty	Moderator	Inparty Strengths vs. Outparty Flaws	0.109	0.121	0.904	0.371	0.831
Moderator	Moderation of Warmth towards Outparty Voters by Frequency Have Conversations with Outparty	Moderator	Inparty Strengths vs. Placebo	-0.098	0.119	-0.824	0.413	0.831
Moderator	Moderation of Warmth towards Outparty Voters by Openness	Moderator	Outparty Flaws vs. Placebo	-0.14	0.119	-1.175	0.245	0.593

Table S4: Numerical Results and Variable Classification: Study 2 (Continued)

Category	Measure Name	DV Type	Condition Comparison	Estimate	SE	t	Unadjusted p-value	Adjusted p-value
Moderator	Moderation of Warmth towards Outparty Voters by Openness	Moderator	Perfect Day vs. Placebo	0.008	0.133	0.062	0.951	1
Moderator	Moderation of Warmth towards Outparty Voters by Openness	Moderator	Inparty Strengths vs. Outparty Flaws	-0.107	0.142	-0.751	0.456	0.831
Moderator	Moderation of Warmth towards Outparty Voters by Openness	Moderator	Inparty Strengths vs. Placebo	-0.246	0.14	-1.758	0.084	0.338
Moderator	Moderation of Warmth towards Outparty Voters by Political Knowledge	Moderator	Outparty Flaws vs. Placebo	-0.143	0.148	-0.962	0.341	0.779
Moderator	Moderation of Warmth towards Outparty Voters by Political Knowledge	Moderator	Perfect Day vs. Placebo	-0.229	0.146	-1.574	0.122	0.465
Moderator	Moderation of Warmth towards Outparty Voters by Political Knowledge	Moderator	Inparty Strengths vs. Outparty Flaws	-0.111	0.19	-0.582	0.564	0.864
Moderator	Moderation of Warmth towards Outparty Voters by Political Knowledge	Moderator	Inparty Strengths vs. Placebo	-0.254	0.2	-1.267	0.211	0.562
Moderator	Moderation of Warmth towards Outparty Voters by Self-Monitoring	Moderator	Outparty Flaws vs. Placebo	-0.101	0.139	-0.727	0.471	0.831

Table S4: Numerical Results and Variable Classification: Study 2 (Continued)

Category	Measure Name	DV Type	Condition Comparison	Estimate	SE	t	Unadjusted p-value	Adjusted p-value
Moderator	Moderation of Warmth towards Outparty Voters by Self-Monitoring	Moderator	Perfect Day vs. Placebo	-0.078	0.16	-0.486	0.629	0.982
Moderator	Moderation of Warmth towards Outparty Voters by Self-Monitoring	Moderator	Inparty Strengths vs. Outparty Flaws	0.143	0.126	1.137	0.261	0.594
Moderator	Moderation of Warmth towards Outparty Voters by Self-Monitoring	Moderator	Inparty Strengths vs. Placebo	0.042	0.124	0.336	0.739	1

Notes: p-values below 0.001 are rounded to 0. As described in the text, p-values for primary outcomes are not adjusted.

Table S5: Means and Standard Deviations of Primary Outcomes, by Condition and Time

(a) Study 1

**Outcome**	**Time Measured**	**Entire Sample**	**Placebo**	**Perfect Day**
Warmth towards Outparty Voters	Baseline	37.29 (25.34)	38.83 (24.74)	35.99 (25.81)
	Immediately After	47.38 (26.5)	44.29 (25.9)	49.98 (26.77)
	3 Months Later	32.76 (24.36)	34.7 (23.55)	31.25 (24.92)
Meta-Perception that Outparty Respects Inparty	Baseline	2.15 (0.82)	2.19 (0.82)	2.13 (0.82)
	Immediately After	2.62 (0.8)	2.44 (0.79)	2.78 (0.78)
	3 Months Later	1.96 (0.76)	1.96 (0.8)	1.97 (0.72)
Choose Candidate Aligned on Issues over Party	Immediately After	0.82 (0.38)	0.84 (0.37)	0.8 (0.4)

Notes: The Table shows the means and standard deviations (in parentheses) for the primary outcome measures at each time. The means at all time points, including baseline, only include participants who were eligible for inclusion in the analysis (e.g., started a conversation).

(b) Study 2

**Primary Outcome**	**Time Measured**	**Entire Sample**	**Placebo**	**Perfect Day**	**Inparty Strengths**	**Outparty Flaws**
Warmth towards Outparty Voters	Baseline	35.23 (22.27)	37.06 (22.75)	30.92 (20.01)	33.06 (21.26)	39.12 (24.05)
	Immediately After	43.53 (24.37)	41.05 (24.62)	46.55 (23.37)	40.38 (25.34)	47.04 (23.61)

Notes: The Table shows the means and standard deviations (in parentheses) for the primary outcome measures at each time. The means at all time points, including baseline, only include participants who were eligible for inclusion in the analysis (e.g., started a conversation).

B Additional recruitment details

B.1 Study 1

After the screener survey, we screened out prospective participants who were not interested in having a conversation or had a suspicious IP address; had bot-like free response questions (as determined by the first author); started the survey more than once (using their participant identification number and their IP address) or did not finish the survey; failed two or more attention checks and identified as a Democrat, or failed one attention check and identified as a Republican; identified as an Independent or did not provide their party identification; or identified as a Democrat and (during some periods when we had a surplus of Democratic participants) did not have a compatible system. Due to an error in the coding, for several waves, we mistakenly invited some Democrat participants who should not have qualified (e.g., because they failed an attention check). We kept these participants.

Among qualifying participants, we assigned participants to time windows when they said they were available (and for those who were not available at any times, we assigned them a random time to give them the option of taking the study); due to an excess of interested Democratic participants, we randomly dropped Democrats when there was a large excess of them available at that time, prioritizing those who were free at that time and had a compatible system.

We sent participants a message through the recruiting platform indicating the day and time the full study would take place, and another reminder right before the study was to take place. (Several days usually elapsed between when participants completed the screener survey and their assigned day for the main conversation survey.)

B.2 Study 2

After the screener survey, we screened out those who were not interested in having a conversation or did not say they were available; identified as an Independent; were Democrats who failed an IP validation logic and failed the compatibility check; or, were Republicans and were not on their phones, had a duplicate IP address or email address, or had an invalid email address. Of these, we assigned participants to a time window they were available. We sent participants an email and text indicating the day and time the conversation survey would take place, and reminded them the day of.

C Relevant previous literature

Given space constraints in the main text, we previous previous research relevant to our study in more detail in this section. Our research builds on other research on the impacts of cross-partisan conversations in several important ways. Most notably, our study is the first to study face-to-face (i.e., video) conversations between outpartisans outside the context of a broader intervention; our study is the first to examine the long-run effects of such contact; and our study is the first to examine a broad variety of outcomes relevant to democratic accountability.

Table S6 reviews existing studies that focus on conversations between outpartisans only, and not combined interventions that include both cross-partisan conversation and other intervention components. Beyond this paper, the only other study to do so is an important working paper by Rossiter, who studies text-based conversations between copartisan strangers (28). Our study builds on Rossiter's work in several ways: we measure effects on outcomes relevant to democratic accountability, we measure attitudinal polarization, and we measure long-run effects. We also examine the effects of conversations more explicitly about partisanship and—inspired by research about the differences between text- and video-based conversations (52)—which take place over video calls.

Table S7 reviews existing studies of interventions that include both conversations with outpartisans and other components. These studies are extremely valuable studies of particular interventions, but cannot speak directly to the effects of cross-partisan conversations because they manipulate both cross-partisan contact and other interventions. A representative example is from Baron and colleagues, who study a day-long depolarization event run by the non-profit Better Angles that involves reflections on intergroup stereotypes, discussions of intergroup bias, a listening exercise, and action-oriented exercises (53). The pathbreaking study by Baron and colleagues is extremely valuable, although it is not focused on cross-partisan conversation per se. Cross-partisan conversation is only one component of the intervention the authors study; the results speak to the effects

of the intervention but leave unclear the effects of just the cross-partisan conversation component. None of the studies in Table S7 study effects on outcomes relevant to democratic accountability, except for one somewhat outcome in Baron and colleagues' work, whether individuals donate to a depolarization NGO.

Table S8 expands our scope to studies of vicarious, imagined, simulated, or reported contact between outpartisans. These are studies that pertain to the impact of cross-partisan conversations but do not randomly assign contact between real outpartisans. For example, (54) study the effects of imagined contact (Study 1) and text-based conversations with confederates (Studies 2 and 3); although (54) produce valuable insights about their research question (the effect of political inclusion in interpersonal conversations), it is unclear whether their findings would generalize to how individuals would react when conversing with an outpartisan who behaves as real people do, instead of how their confederates did. None of these studies examine long-run effects, effects on attitudinal polarization, nor effects on democratic accountability.

Finally, Table S9 provides a non-exhaustive list of examples of other studies that examine effects of various interventions related to the topics of either conversations or affective polarization, but do not concern the effects of cross-partisan conversations on affective polarization.

Table S6: Studies of Conversations with Outpartisans

	Research Design			Measures...		
Citation	Outpartisan Conversation Treatment(s)	Additional Components of Intervention	Conversation Format	Democratic Accountability?	Intergroup Attitudes?	Long-Run Effects?
This paper	Conversation with an outpartisan about perfect day; Conversation with an outpartisan about areas of disagreement	-	Video conversation with a stranger	Yes (e.g., Party Loyalty, Democratic Norms, Bipartisanship)	Yes	Yes, approx. three months post-intervention
Rossiter	Non-political conversation with an outpartisan; Conversation with an outpartisan about an issue (gun control)	-	Text-based conversation with a stranger	-	Yes	-

Table S7: Studies of Interventions that Include Both Conversations with Outpartisans and Other Components

	Research Design			Measures...		
Citation	Outpartisan Conversation Treatment(s)	Additional Components of Intervention	Conversation Format	Democratic Accountability?	Intergroup Attitudes?	Long-Run Effects?
(55)	Politically heterogenous discussion group (vs. homogenous discussion or a no-contact control)	Ambivalent vs univalent partisan prime; All members read about policy	In-person in groups	-	-	-
(15)	Heterogeneous political discussion group about commonalities between parties (vs. homogenous political discussion group or non-political discussion group)	Heterogenous group reads article about common ground between parties; homogenous group reads article about partisan division and polarization; Non-political discussion group reads article about beach towns	In-person in groups	-	Yes	Yes, one week later
(56)	Implementing door-to-door persuasion campaigns; targeted voters include outpartisans	Training and implementation of two-way narrative exchanges, perspective-getting exercises	In-person, door-to-door	-	Yes	Only measure is approx. one week later
(35)	Work in heterogenous groups to devise questions for experts and discuss political issues in five areas as part of deliberation event (vs. pure control)	Cooperation to devise questions; expert testimony; briefing materials with evidence for against/proposals; Attendance at deliberation event	In-person in groups	-	Yes	-
(53)	Guided heterogeneous group discussion as part of reciprocal group reflection exercises	Depolarization workshop, including reflections on stereotypes, listening exercise, action-oriented exercises	In-person, as part of depolarization workshop	Yes (donation to a depolarization NGO)	Yes	Yes, six months

Table S8: Vicarious, imagined, simulated or reported contact between outpartisans

Citation	Research Design			Measures...		
	Outpartisan Conversation Treatment(s)	Additional Components of Intervention	Conversation Format	Democratic Accountability?	Intergroup Attitudes?	Long-Run Effects?
(54)	Imagined contact (Study 1); Conversations with confederates (Studies 2 and 3)	Participants excluded or included by outpartisans	Imagined contact (Study 1); Text-based conversations with confederates (Studies 2 and 3)	-	Yes	-
(57)	Read about or imagine contact an outparty member (vs. inparty or control)	Contact type is positive / cooperative, negative, or neutral (studies 2 and 3)	Reported (Study 1), vicarious (Study 2) or imagined contact (Study 3)	-	Yes	-
(58)	Imagined contact with outpartisans	Valence of contact (positive vs. negative)	Imagine contact	-	Yes	-
(23)	Self-reported contact (not randomized; observational study)	n/a	Self-reported participation in online and face-to-face heterogenous discussions	-	Yes	-

Table S9: Examples of other studies that do not examine effects of contact between partisan groups

Citation	Research Design			Measures...		
	Outpartisan Conversation Treatment(s)	Additional Components of Intervention	Conversation Format	Democratic Accountability?	Intergroup Attitudes?	Long-Run Effects?
(59, Study 5)	Conversation with confederate who disagrees on gun issues; no control without a conversation	Confederate shares personal experience or fact	In-person conversation with a confederate	-	-	-
(7)	None	Common ingroup identity prime	n/a	-	Yes	-
(60)	None	Exposure to outpartisan politicians' messages on Twitter	n/a	-	-	-

REFERENCES AND NOTES

1. S. Iyengar, G. Sood, Y. Lelkes, Affect, not ideology a social identity perspective on polarization. *Public Opin. Q.* **76**, 405–431 (2012).

2. M. K. Chen, R. Rohla, The effect of partisanship and political advertising on close family ties. *Science* **360**, 1020–1024 (2018).

3. G. A. Huber, N. Malhotra, Political homophily in social relationships: Evidence from online dating behavior. *J. Polit.* **79**, 269–283 (2017).

4. C. M. Connell, Y. Margalit, N. Malhotra, M. Levendusky, The economic consequences of partisanship in a polarized era. *Am. J. Polit. Sci.* **62**, 5–18 (2018).

5. P. Pierson, E. Schickler, Madison's constitution under stress: A developmental analysis of political polarization. *Annu. Rev. Polit. Sci.* **23**, 37–58 (2020).

6. S. Iyengar, Y. Lelkes, M. Levendusky, N. Malhotra, S. J. Westwood, The origins and consequences of affective polarization in the united states. *Annu. Rev. Polit. Sci.* **22**, 129–146 (2019).

7. M. S. Levendusky, Americans, not partisans: Can priming american national identity reduce affective polarization? *J. Polit.* **80**, 59–70 (2018).

8. N. Bowles, "How to get trump voters and liberals to talk: Don't make anyone sit in a circle," *New York Times*, 2019.

9. L. Mason, "Mandatory national service," *POLITICO*, 2019.

10. E. L. Paluck, S. A. Green, D. P. Green, The contact hypothesis re-evaluated. *Behav. Public Policy* **3**, 129–158 (2019).

11. E. L. Paluck, Is it better not to talk? group polarization, extended contact, and perspective taking in eastern democratic Republic of Congo. *Pers. Soc. Psychol. Bull.* **36**, 1170–1185 (2010).

12. E. L. Paluck, R. Porat, C. S. Clark, D. P. Green, Prejudice reduction: Progress and challenges. *Annu. Rev. Psychol.* **72**, 533–560 (2021).

13. D. J. Ahler, Self-fulfilling misperceptions of public polarization. *J. Polit.* **76**, 607–620 (2014).

14. D. J. Ahler, G. Sood, The parties in our heads: Misperceptions about party composition and their consequences. *J. Polit.* **80**, 964–981 (2018).

15. M. Levendusky, D. Stecula, *We Need to Talk: How Cross-Party Dialogue Reduces Affective Polarization* (Cambridge Univ. Press, 2021).

16. L. V. Orr, G. A. Huber, The policy basis of measured partisan animosity in the United States. *Am. J. Pol. Sci.* **64**, 569–586 (2020).

17. D. Broockman, J. Kalla, Durably reducing transphobia: A field experiment on door-to-door canvassing. *Science* **352**, 220–224 (2016).

18. G. W. Allport, *The Nature of Prejudice* (Addison-Wesley, 1954).

19. T. F. Pettigrew, L. Tropp, A meta-analytic test of intergroup contact theory. *J. Pers. Soc. Psychol.* **90**, 751–783 (2006).

20. B. R. Warner, H. K. Horstman, C. C. Kearney, Reducing political polarization through narrative writing. *J. Appl. Commun. Res.* **48**, 459–477 (2020).

21. R. D. Enos, Causal effect of intergroup contact on exclusionary attitudes. *Proc. Natl. Acad. Sci. U.S.A.* **111**, 3699–3704 (2014).

22. C. C. MacInnis, E. Page-Gould, How can intergroup interaction be bad if intergroup contact is good? Exploring and reconciling an apparent paradox in the science of intergroup relations. *Perspect. Psychol. Sci.* **10**, 307–327 (2015).

23. E. Amsalem, E. Merkley, P. J. Loewen, Does talking to the other side reduce inter-party hostility? evidence from three studies. *Political Commun.* **39**, 61–78 (2021).

24. A. Aron, E. Melinat, E. N. Aron, R. D. Vallone, R. J. Bator, The experimental generation of interpersonal closeness: A procedure and some preliminary findings. *Pers. Soc. Psychol. Bull.* **23**, 363–377 (1997).

25. D. E. Broockman, J. L. Kalla, S. Westwood, Does affective polarization undermine democratic norms or accountability? Maybe not. *Am. J. Polit. Sci.* (2022).

26. J. N. Druckman, M. S. Levendusky, What do we measure when we measure affective polarization? *Public Opin. Q.* **83**, 114–122 (2019).

27. A. Coppock, *Persuasion in Parallel* (Chicago Studies in American Politics, University of Chicago Press, 2022, Forthcoming).

28. E. Rossiter, The consequences of interparty conversation on outparty affect and stereotypes, paper presented at the 2020 Meeting of the Society for Political Methodology, 2020.

29. A. S. Gerber, D. P. Green, *Field Experiments: Design, Analysis, and Interpretation* (WW Norton, 2012).

30. M. L. Anderson, Multiple inference and gender differences in the effects of early intervention: A reevaluation of the Abecedarian, Perry preschool, and early training projects. *J. Am. Stat. Assoc.* **103**, 1481–1495 (2008).

31. J. G. Bullock, D. P. Green, S. E. Ha, Yes, but what's the mechanism? (don't expect an easy answer). *J. Pers. Soc. Psychol.* **98**, 550–558 (2010).

32. J. A. Minson, F. S. Chen, C. H. Tinsley, Why won't you listen to me? Measuring receptiveness to opposing views. *Manage. Sci.* **66**, 3069–3094 (2020).

33. S. Klar, Y. Krupnikov, *Independent Politics* (Cambridge Univ. Press, 2016).

34. J. N. Druckman, M. J. Kifer, M. Parkin, Campaign rhetoric and the incumbency advantage. *Am. Politics Res.* **48**, 22–43 (2020).

35. J. Fishkin, A. Siu, L. Diamond, N. Bradburn, Is deliberation an antidote to extreme partisan polarization? Reflections on “America in one room”. *Am. Polit. Sci. Rev.* **115**, 1464–1481 (2021).

36. D. C. Mutz, Cross-cutting social networks: Testing democratic theory in practice. *Am. Polit. Sci. Rev.* **96**, 111–126 (2002).

37. G. Itzchakov, N. Weinstein, N. Legate, M. Amar, Can high quality listening predict lower speakers’ prejudiced attitudes? *J. Exp. Soc. Psychol.* **91**, 104022 (2020).

38. M. Yeomans, M. E. Schweitzer, A. W. Brooks, The conversational circumplex: Identifying, prioritizing, and pursuing informational and relational motives in conversation. *Curr. Opin. Psychol.* **44**, 293–302 (2022).

39. M. Yeomans, J. Minson, H. Collins, F. Chen, F. Gino. Conversational receptiveness: Improving engagement with opposing views. *Organ. Behav. Hum. Decis. Process.* **160**, 131–148 (2020).

40. J. Parkinson, J. Mansbridge, *Deliberative Systems: Deliberative Democracy at the Large Scale* (Cambridge Univ. Press, 2012).

41. J. Kalla, D. Broockman, Which narrative strategies durably reduce prejudice? Evidence from field and survey experiments supporting the efficacy of perspective-getting. *Am. J. Polit. Sci.* (2021).

42. J. Lees, M. Cikara, Inaccurate group meta-perceptions drive negative out-group attributions in competitive contexts. *Nat. Hum. Behav.* **4**, 279–286 (2020).

43. N. Kteily, E. Bruneau, A. Waytz, S. Cotterill, The ascent of man: Theoretical and empirical evidence for blatant dehumanization. *J. Pers. Soc. Psychol.* **1090**, 901 (2015).

44. J. L. Martherus, A. G. Martinez, P. K. Piff, A. G. Theodoridis, Party animals? extreme partisan polarization and dehumanization. *Polit. Behav.* **43**, 517–540 (2021).

45. A. T. Little, K. Schnakenberg, I. R. Turner, Motivated reasoning and democratic accountability. *Am. Political Sci. Rev.* **116**, 751–767 (2022).

46. L. Harbridge, N. Malhotra, Electoral incentives and partisan conflict in congress: Evidence from survey experiments. *Am. J. Polit. Sci.* **55**, 494–510 (2011).

47. J. N. Druckman, K. R. Nelson, Framing and deliberation: How citizens' conversations limit elite influence. *Am. J. Pol. Sci.* **47**, 729–745 (2003).

48. J. R. Zaller, *The Nature and Origins of Mass Opinion* (Cambridge Univ. Press, 1992).

49. S. D. Gosling, P. J. Rentfrow, W. B. Swann Jr., A very brief measure of the big-five personality domains. *J. Res. Pers.* **37**, 504–528 (2003).

50. M. Snyder, Self-monitoring of expressive behavior. *J. Pers. Soc. Psychol.* **30**, 526–537 (1974).

51. D. Watson, L. A. Clark, A. Tellegen, Development and validation of brief measures of positive and negative affect: The PANAS scales. *J. Pers. Soc. Psychol.* **54**, 1063–1070 (1988).

52. J. Schroeder, M. Kardas, N. Epley, The humanizing voice: Speech reveals, and text conceals, a more thoughtful mind in the midst of disagreement. *Psychol. Sci.* **28**, 1745–1762 (2017).

53. H. Baron, R. Blair, D. D. Choi, L. Gamboa, J. Gottlieb, A. L. Robinson, S. Rosenzweig, M. Turnbull, E. A. West, Can Americans depolarize? Assessing the effects of reciprocal group reflection on partisan polarization, paper presented at the Evidence in Governance in Politics Series, 2022.

54. J. G. Voelkel, D. Ren, M. J. Brandt Inclusion reduces political prejudice. *J. Exp. Soc. Psychol.* **95**, 104149 (2021).

55. S. Klar, Partisanship in a social setting. *Am. J. Polit. Sci.* **58**, 687–704 (2014).

56. J. L. Kalla, D. E. Broockman, *Voter Outreach Campaigns Can Reduce Affective Polarization Among Implementing Political Activists* (American Political Science Review, 2022).

57. M. Wojcieszak, B. R. Warner, Can interparty contact reduce affective polarization? A systematic test of different forms of intergroup contact. *Polit. Commun.* **37**, 789–811 (2020).

58. B. R. Warner, A. Villamil, A test of imagined contact as a means to improve cross-partisan feelings and reduce attribution of malevolence and acceptance of political violence. *Commun. Monogr.* **84**, 447–465 (2017).

59. E. Kubin, C. Puryear, C. Schein, K. Gray, Personal experiences bridge moral and political divides better than facts. *Proc. Natl. Acad. Sci. U.S.A.* **118**, e2008389118 (2021).

60. C. A. Bail, L. P. Argyle, T. W. Brown, J. P. Bumpus, H. Chen, M. B. F. Hunzaker, J. Lee, M. Mann, F. Merhout, A. Volfovsky, Exposure to opposing views on social media can increase political polarization. *Proc. Natl. Acad. Sci. U.S.A.* **115**, 9216–9221 (2018).
